# Targeted Proteomics Approach Toward Understanding the Role of the Mitochondrial Protease FTSH4 in the Biogenesis of OXPHOS During Arabidopsis Seed Germination

**DOI:** 10.3389/fpls.2018.00821

**Published:** 2018-06-15

**Authors:** Malgorzata Heidorn-Czarna, Dominik Domanski, Malgorzata Kwasniak-Owczarek, Hanna Janska

**Affiliations:** ^1^Department of Cellular Molecular Biology, Faculty of Biotechnology, University of Wrocław, Wrocław, Poland; ^2^Mass Spectrometry Laboratory, Institute of Biochemistry and Biophysics, Polish Academy of Sciences, Warsaw, Poland

**Keywords:** targeted proteomics, multiple reaction monitoring (MRM), Arabidopsis, germination, mitochondria, ATP-dependent proteases, FTSH4 protease

## Abstract

Seed germination provides an excellent model to study the process of mitochondrial biogenesis. It is a complex and strictly regulated process which requires a proper biogenesis of fully active organelles from existing promitochondrial structures. We have previously reported that the lack of the inner mitochondrial membrane protease FTSH4 delayed Arabidopsis seed germination. Here, we implemented a targeted mass spectrometry-based approach, Multiple Reaction Monitoring (MRM), with stable-isotope-labeled standard peptides for increased sensitivity, to quantify mitochondrial proteins in dry and germinating wild-type and *ftsh4* mutant seeds, lacking the FTSH4 protease. Using total seed protein extracts we measured the abundance of the peptide targets belonging to the OXPHOS complexes, AOX1A, transport, and inner membrane scaffold as well as mitochondrial proteins that are highly specific to dry and germinating seeds. The MRM assay showed that the abundance of these proteins in *ftsh4* did not differ substantially from that observed in wild-type at the level of dry seed and after stratification, but we observed a reduction in protein abundance in most of the examined OXPHOS subunits in the later stages of germination. These changes in OXPHOS protein levels in *ftsh4* mutants were accompanied by a lower cytochrome pathway activity as well as an increased AOX1A amount at the transcript and protein level and alternative pathway activity. The analyses of the steady-state transcript levels of mitochondrial and nuclear genes encoding OXPHOS subunits did not show significant difference in their amount, indicating that the observed changes in the OXPHOS occurred at the post-transcriptional level. At the time when *ftsh4* seeds were fully germinated, the abundance of the OXPHOS proteins in the mutant was either slightly lowered or comparable to these amounts in wild-type seeds at the similar developmental stage. By the implementation of an integrative approach combining targeted proteomics, quantitative transcriptomics, and physiological studies we have shown that the FTSH4 protease has an important role in the biogenesis of OXPHOS and thus biogenesis of mitochondria during germination of Arabidopsis seeds.

## Introduction

In the plant life cycle, seed germination has been recognized as one of the most critical phases, in which structurally simple and metabolically quiescent organelles in dry embryos develop into mature functional forms to support the more energy-demanding processes of cell division and organogenesis of the new seedling (Paszkiewicz et al., 2017). Germination *sensu stricto*, which portrays physical and metabolic processes occurring in imbibed seeds, begins with the uptake of water by the dry seed and ends with the emergence of the radicle, being a visible symptom of the completion of germination (Rajjou et al., 2012; Czarna et al., 2016).

Mitochondrial biogenesis is often described as the change from dormant promitochondria to metabolically and energetically active mature mitochondria, and the reactivation of mitochondrial bioenergetics is a key process during germination (Carrie et al., 2013). This process includes biogenesis of mitochondrial membranes, especially the inner membrane with numerous cristae and extensive compartmentalization, as well as synthesis and assembly of mitochondrial protein complexes (Paszkiewicz et al., 2017). Upon an in-depth examination of gene expression studied mainly at the transcript level a model illustrating sequential and dynamic molecular aspects of mitochondrial biogenesis during *Arabidopsis thaliana* seed germination was established (Narsai et al., 2011; Law et al., 2012). It starts with a transient burst in the expression of genes encoding mitochondrial proteins involved in transcription and RNA metabolism, followed by the expression of genes involved in replication and translation of the mitochondrial genome (Law et al., 2012; Carrie et al., 2013). Next, a peak in the abundance of transcripts associated with protein import occurs, followed by the progressive accumulation of transcripts for nucleus-encoded components of the electron transport chain. Although there have been several extensive studies published in the last few years, which are associated with transcriptomic changes in seed mitochondria (Narsai et al., 2011, 2017; Law et al., 2012), there is relatively little information addressing the detailed time course of mitochondrial protein dynamics in germinating seeds. Until now, several studies using gel-based [two-dimensional (2-D) gel electrophoresis, western blotting] or gel-free proteomic approaches [mass spectrometry-based isobaric tag for relative and absolute quantitation (iTRAQ) or shotgun proteomics] have been carried out, which showed the differential abundance of various mitochondrial proteins in whole germinating seeds or isolated organelles of different plant species (e.g., *A. thaliana, Oryza sativa, Pisum sativum, Zea mays*) (Logan et al., 2001; Howell et al., 2006, 2007; Taylor et al., 2010; Law et al., 2012; Galland et al., 2014; Han et al., 2014; reviewed in Czarna et al., 2016). No detailed proteomics surveys have been applied so far to examine the biogenesis of the oxidative phosphorylation (OXPHOS) complexes throughout the seed germination course.

Proper biogenesis of the OXPHOS machinery requires strict coordination of gene expression of both nuclear and mitochondrial genomes and must be monitored by molecular chaperones and proteases (Baker and Haynes, 2011; Welchen et al., 2014). Evidence for the participation of the mitochondrial ATP-dependent proteases in the biogenesis of mitochondria comes from studies of different organisms (Janska et al., 2010). These enzymes are unique since they combine two opposite activities: proteolytic and chaperone-like. Our studies revealed that *A. thaliana* devoid of FTSH4, one of the mitochondrial inner membrane ATP-dependent proteases, displays a delay in seed germination (Gibala et al., 2009), however, to date no further in-depth research has been implemented to characterize molecular bases of this dysfunction and, in consequence, to define the role of FTSH4 in Arabidopsis seeds. So far, the function of the FTSH4 protease was extensively studied only during the post*-*germination growth phase and largely under stress conditions (Kolodziejczak et al., 2007; Gibala et al., 2009; Kicia et al., 2010; Zhang et al., 2014a,b; Dolzblasz et al., 2016; Hong et al., 2016; Smakowska et al., 2016; Opalinska et al., 2017a,b; Zhang et al., 2017). We found that a lack of FTSH4 leads to oxidative stress, a decreased activity and abundance of mitochondrial complexes I and V, a lowered amount of cardiolipin, and an alteration in protein import through the inner membrane translocase TIM17:23. In addition, one of the characteristics of the *ftsh4* mutant is the presence of giant, spherical mitochondria coexisting among normal ones (Smakowska et al., 2016).

The recent development of a mass spectrometry-based targeted proteomics approach, such as *Multiple Reaction Monitoring* (MRM: the acquisition of multiple product ions from one precursor ion by Selected Reaction Monitoring (SRM) transitions), and its utilization for the quantitative analysis of peptides derived from proteins of specific organelles in plant cells has become a powerful, hypothesis-driven analytical tool to achieve specific, sensitive, and precise protein quantification (Zulak et al., 2009; Hall et al., 2011; Fan et al., 2012; Hooper et al., 2017). The high specificity of MRM analysis comes from the mass measurement (accurately measured to an atomic mass unit) of the whole peptide and its multiple fragments, the unique peptide ion fragmentation pattern, and the precise peptide retention time in the liquid chromatography (LC) separation (Liebler and Zimmerman, 2013). During MRM analysis of samples with internally spiked stable isotope-labeled standard (SIS) peptides, these parameters are always compared between the potentially detected endogenous peptide and the corresponding standard peptide, and all need to match. This gives MRM analysis near-perfect molecular discrimination and allows for precise normalization and quantitation of peptide amounts as well as to differentiate between protein isoforms (Zulak et al., 2009; Bru-Martínez et al., 2018).

Over the last few years, MRM has been successfully implemented in plant subcellular proteomic studies, such as on mitochondria isolated from different tissues, which included large-sized plant seeds like rice (Taylor et al., 2010, 2014; Huang et al., 2013). For small-sized seeds, such as from Arabidopsis, however, where the isolation and enrichment of organelles is practically impossible, targeted subcellular proteomic methods to quantitate specifically targeted organellar proteins have never been used before. In the present work, we demonstrate for the first time the use of MRM to measure the abundance of the OXPHOS subunits (NADH dehydrogenase iron-sulfur protein 1, NAD75; NADH dehydrogenase iron-sulfur protein 2, NAD7; gamma carbonic anhydrase 2, CA2; succinate dehydrogenase flavoprotein subunit 1, SDH1-1; succinate dehydrogenase iron-sulfur subunit 2, SDH2-2; cytochrome *b*, COB; cytochrome *b-c1* complex subunit Rieske-2, RIESKE; cytochrome *c* oxidase subunit 2, COX2; cytochrome *c* oxidase subunit 5C-1, COX5C-1; ATP synthase subunit alpha, ATP1; ATP synthase subunit beta-1, ATP2-1), alternative oxidase 1A (AOX1A), transport (mitochondrial outer membrane voltage-dependent anion channel 1 and 3, VDAC1 and VDAC3) and inner membrane scaffold (prohibitin 3, PHB3) proteins as well as mitochondrial proteins that are highly specific to dry and germinating seeds (succinate dehydrogenase iron-sulfur subunit 3, SDH2-3; alternative oxidase 2, AOX2; cardiolipin synthase, CLS) in a complex Arabidopsis seed protein sample. By applying proteomic and transcriptomic tools, targeted at the selected mitochondrial proteins, we show that the loss of the FTSH4 protease disturbs the biogenesis of the OXPHOS complexes, leading to a delay in biogenesis of mitochondria, which correlates with delay in germination of *ftsh4* seeds. This proteomic study provides an insight into mitochondrial biogenesis during plant germination as well as into the involvement of the mitochondrial ATP-dependent proteases in that process.

## Materials and methods

### Plant material and growth conditions

All *A. thaliana* plants used in this study were of the Columbia-0 ecotype (Col-0). The transgenic lines *ftsh4-1* (SALK_035107/TAIR) and *ftsh4-2* (GABI_103H09/TAIR) were purchased from the Salk Institute and the Max Planck Institute for Breeding Research, respectively. The lines were previously characterized in Gibala et al. (2009) and Smakowska et al. (2016). Plants were grown in soil in a 16-h light/8-h dark (long-day, LD) photoperiod at 22°C, with a light intensity of 150 μmol m^−2^ s^−1^ and 70% humidity. Fully developed and ripened brown siliques of the wild-type and *ftsh4* mutant plants were collected at the same time point.

### Seed germination assay

The studied seeds were surface sterilized by washing in 70% (v/v) ethanol for 5 min and then in 5% (v/v) household bleach for 10 min with mild rotation, followed by several rinses in sterile water. The sterilized seeds were then spread on a black nitrocellulose membrane with a white grid (ME 25/31 ST, 47 mm diameter, GE Healthcare) over two circles of Whatman filter paper (3MM CHR, GE Healthcare) in 8-cm Petri dish wetted with sterile distilled water and stratified for 48 h at 4°C and in the dark. After stratification, the plates were transferred to a growth chamber and seeds were incubated at 22°C or 30°C under long-day photoperiod with a light intensity of 150 μmol m^−2^ s^−1^. Germination rate was determined on at least four biological replicates of 100–200 seeds of each type. Seeds were scored every several hours until seed germination rate reached over 98%. A seed was considered as germinated after the emergence of the radical tip through the seed coat.

### Seed collection for proteomic, transcript, and respiration analyses

For proteomic analysis, 75 mg of surface sterilized wild-type and *ftsh4* seeds were collected at the following time points: dry seeds (DS, ~3-months-old), seeds after 48 h of stratification (4°C, in the dark) and no germination (0 h), seeds after 48 h stratification followed by germination at 30°C and LD photoperiod for 6, 12, 24, and 42 h (6, 12, 24, and 42 h). For real-time PCR analysis, the samples were collected from the same time points except for 42 h. For oxygen consumption measurements, the sampling of 10-20 mg of seeds was carried out at 0h, 6h, 12h, 24h, and 42h. Stratification and germination were performed on two circles of Whatman paper in 8-cm Petri dish wetted with sterile distilled water.

### Total protein extraction from dry and germinating seeds

Total protein extracts were obtained from 75 mg of dry mature seeds and seeds collected at a given time point of the germination course (0, 6, 12, 24, and 42 h), as described before (Rajjou et al., 2006), with some modifications. Seeds were ground in liquid nitrogen using mortar and pestle and total proteins were extracted at 4°C in 1 ml of lysis buffer [8 M urea, 2 M thiourea, 4% (w/v) CHAPS, 30 mM Tris, pH 8.5, the protease inhibitor cocktail Complete Mini (Roche Diagnostics GmbH), 53 U/ml DNase I (Thermo Fisher Scientific), 5 Kunitz units/ml RNase A (Eurx), 20 mM DTT and 0.2% Trition X-100 (v/v)] with intensive stirring for 30 min followed by centrifugation at 30,000 × g, 4°C, for 10 min. The obtained supernatant was subjected to another centrifugation as above. The final supernatant constituted total seed protein extract. Protein concentrations in wild-type and *ftsh4* seed extracts were determined using the RC DC Protein Assay (Bio-Rad).

### Multiple reaction monitoring (MRM)

#### Seed protein sample fractionation

**Figure 1:**
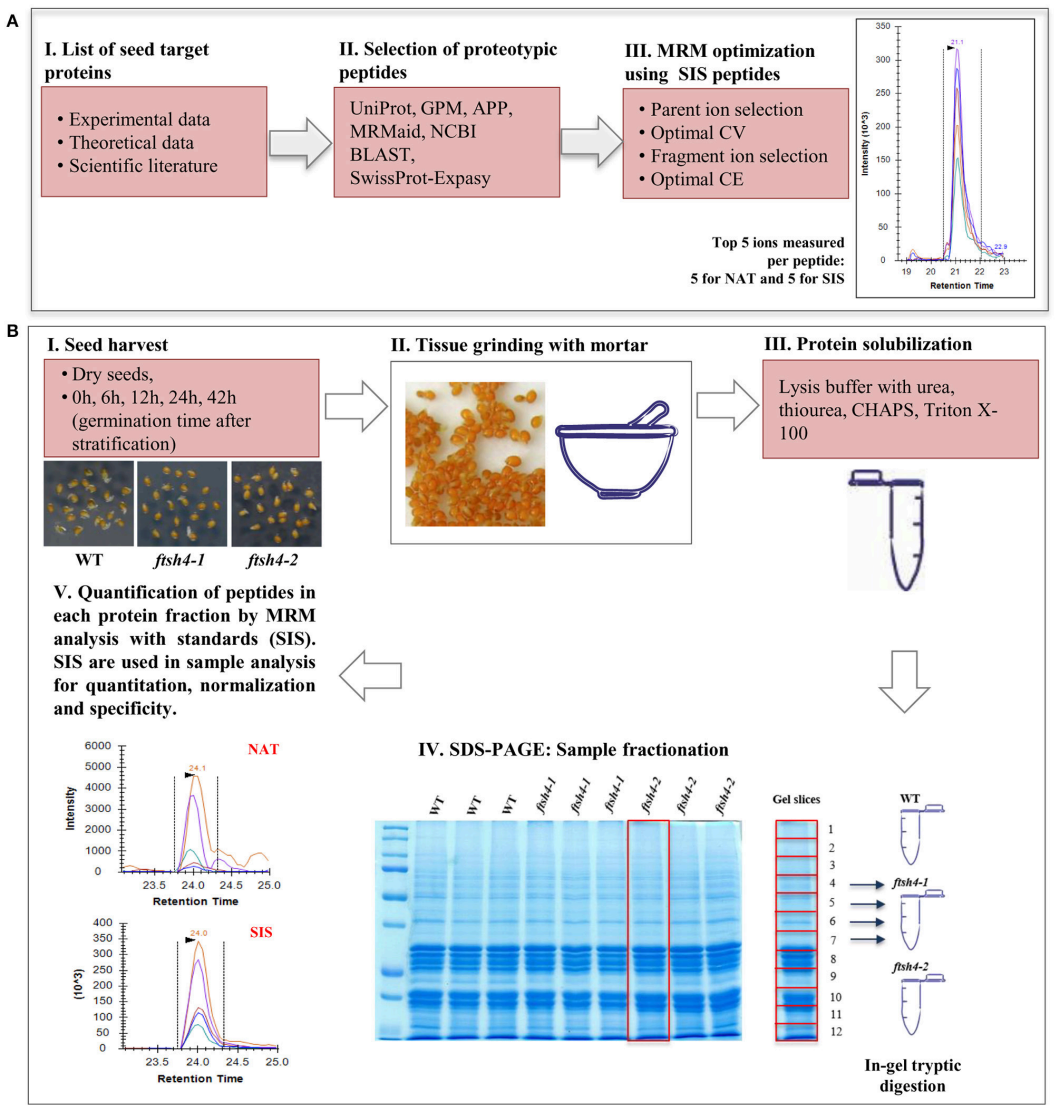
Schematic experimental workflow of the MRM assay of Arabidopsis seed mitochondrial proteins. **(A)** Selection of proteins of interest, design of the peptides specific for the target protein and optimization of the MRM method. **(B)** MRM analysis of seed mitochondrial proteins: I, II, III—collection of the seed samples from wild-type and *ftsh4* mutants, protein extraction and solubilization. IV, Fractionation of the total seed protein extracts (50 μg) of WT, *ftsh4-1* and *ftsh4-2* from each studied stage (DS, 0, 6, 12, 24, and 42 h) via 12% SDS-PAGE. Preparation of the samples for quantitative mass-spectrometry analysis. V, Quantification of the selected seed mitochondrial proteins by MRM peptide analysis with standards (SIS). CV, cone voltage; CE, collision energy; NAT, natural peptide; SIS, stable-isotope labeled standard peptide analog. More details on the survey design of the experiment are given in the main text of the section Materials and Methods as well as Results sections.

Total seed protein extracts obtained from three biological replicates of dry seeds and seeds collected from every time of germination of wild-type and *ftsh4* mutants were precipitated with the ReadyPrep 2-D Cleanup kit (Bio-Rad). Proteins were quantified with the RC DC Protein Assay reagents (Bio-Rad). Fifty microgram aliquots of the total seed protein extract were resuspended in Laemmli Sample Buffer (Bio-Rad) with 2-mercaptoethanol and boiled for 10 min. Samples were resolved on sodium dodecyl sulfate polyacrylamide gel electrophoresis (SDS-PAGE) using 1 mm, 12% polyacrylamide gels. Proteins were fixed in fixing solution (40% (v/v) methanol, 10% (v/v) acetic acid) and visualized by staining gels with colloidal Coomassie solution (0.02% (w/v) CBB G-250, 5% (w/v) aluminum sulfate, 10% (v/v) methanol, 2% (v/v) orthophosphoric acid). The SDS-PAGE gels were then destained with destaining solution (10% (v/v) methanol, 2% (v/v) orthophosphoric acid). Gel lines each representing an individual biological replicate of a studied sample were manually excised and divided into 12 equivalent slices (Figure 1, Figure S1).

#### Protein digestion

Excised gel bands were in-gel digested using the method of Shevchenko et al. (2006) with minor modifications as follows. In brief, the gel pieces were rinsed in 100 mM ammonium bicarbonate (AmBic) and dried with neat acetonitrile. Reduction was performed by adding 50 μl of 10 mM tris(2-carboxyethyl)phosphine hydrochloride (TCEP) (in 100 mM AmBic) and incubating at 60°C for 30 min. After drying the gel again with acetonitrile, alkylation was performed by adding 50 μl of 50 mM iodoacetamide (in 100 mM AmBic) and incubating for 20 min at room temperature in the dark. The gel pieces were then dried with acetonitrile, de-stained in a 1:1 mixture of 100 mM AmBic and acetonitrile and dried again with acetonitrile. Gel pieces were then rehydrated for 2 h at 4°C after adding 60 μl of sequencing-grade trypsin (Promega) (0.8 μg in 10 mM AmBic, 10% acetonitrile). After adding another 20 μl of 10 mM AmBic, 10% acetonitrile, digestion was carried on for 6 h at 37°C. Peptides were extracted with 160 μl of a 1:2 solution of 5% formic acid/acetonitrile. A mixture of SIS peptides was added to give an amount of 2.5 pmol of each SIS peptide per total extract. Samples were subsequently dried down in a vacuum centrifuge, re-dissolved with 25 μl of 0.1% formic acid in a sonication bath for 5 min, bench-top centrifuged (12,000 × g, 10 min), transferred to high performance liquid chromatography (HPLC) auto-sampler vials, and 3.8 μl was used for nano-LC-MRM analysis.

#### Selection of peptides for MRM analysis

Peptides for the MRM protein panel were selected with the following criteria and using tools and databases such as: UniProt, Global Proteome Machine (GPM), Arabidopsis Proteotypic Predictor (APP, Taylor et al., 2014), MRMaid (Fan et al., 2012), SwissProt-Expasy, and NCBI BLASTp. The length of each peptide did not exceed 20 amino acids and easily chemically modifiable residues of C, M, W, Q on the N-terminal end as well as sequences of DP, DG, NG or QG were avoided if possible (Table 1). Selected peptides were characterized by a high number of observations in the GPM and PRIDE (through MRMaid) spectral library databases or by high probability of observation according to the APP if no spectral observations were reported. Peptide uniqueness was checked by NCBI BLASTp and through APP and MRMaid. Predicted trypsin efficiency was checked using the SwissProt-Expasy PeptideCutter. Peptides were also checked to avoid those with post-translational modifications, molecular processing or presence of single-nucleotide polymorphisms (SNPs) in UniProt.

**Table 1:** Selected mitochondrial proteins and their peptides chosen for MRM analysis using total protein of wild-type and *ftsh4* mutant seeds.

**AGI**	**Protein**	**Sequence**	**Sequence localization**	
			**Start**	**End**
**COMPLEX I SUBUNITS**				
AtMg00510	NADH dehydrogenase iron-sulfur protein 2, NAD7	LVDIGTVTAQQAK	203	215
		DIDSFTQQFASR	174	185
		GEFGVFLVSNGSNRPYR	336	352
		LLEFYER	141	147
At5g37510	NADH dehydrogenase iron-sulfur protein 1, NAD75	FASEVAGVQDLGILGR	227	242
		NPAIIVGAGLFNR	511	523
		VHFSNPEDAIEVFVDGYAVK	65	84
		LNEDINEEWISDK	319	331
At1g47260	Gamma carbonic anhydrase 2, CA2	NYINLAQIHASENSK	206	220
		SFEQIEVER	221	229
		AIYTVGNWIR	7	16
		LTDEEIVYISQSAK	192	205
**COMPLEX II SUBUNITS**				
At5g66760	Succinate dehydrogenase flavoprotein subunit 1, SDH1-1	AFGGQSLDFGK	160	170
		SSQTILATGGYGR	238	250
		NSNGSLPTSTIR	491	502
		LPGISETAAIFAGVDVTK	369	386
At5g40650	Succinate dehydrogenase iron-sulfur subunit 2, SDH2-2	WNPDNPGKPELQDYK	56	70
		ETTITPLPHMFVIK	131	144
		ASTGGGGASLK	39	49
		NPASVPGK	168	175
At5g65165	Succinate dehydrogenase iron-sulfur subunit 3, SDH2-3	HLLSDPLVR	297	305
		DLVVDLTNFYQQYK	165	178
		GLNPASAILK	283	292
		LQAITESETK	257	266
**COMPLEX III SUBUNITS**				
AtMg00220	Cytochrome *b*, COB	DVEGGWLLR	77	85
		IAFYPYFYVK	225	234
		GLY**Y**ASYSSPR^*^	106	116
		GLY**H**ASYSSPR^**^	106	116
At5g13440	Cytochrome *b-c1* complex subunit Rieske-2, RIESKE	AFAYFVLSGGR	112	122
		IVYDDHNHER^#^	93	102
		GPAPYNLEVPTYSFLEENK	252	270
		FVYASVLR	123	130
**COMPLEX IV SUBUNITS**				
AtMg00160	Cytochrome *c* oxidase subunit 2, COX2	LNQISILVQR	205	214
		LLEVDNR	161	167
		IIVTSADV**L**HSWAVPS**L**GVK^*^	178	197
		IIVTSADV**P**HSWAVPS**S**GVK^**^	178	197
At2g47380	Cytochrome *c* oxidase subunit 5C-1, COX5C-1	TFYDLLER	47	54
		ELFIGLALGLAAGGLWK^#^	19	35
		VAHATLK^#^	6	12
**ATP SYNTHASE SUBUNITS**				
AtMg01190	ATP synthase subunit alpha, mitochondrial, ATP1	AVDSLVPIGR	154	163
		TTIAIDTILNQK	178	189
		EAFPGDVFYLHSR	295	307
		GIRPAINVGLSVSR	363	376
At5g08670	ATP synthase subunit beta-1, mitochondrial, ATP2-1	DAEGQDVLLFIDNIFR	322	337
		FTQANSEVSALLGR	338	351
		DAPALVDLATGQEILATGIK	194	213
		VGLTGLTVAEYFR	309	321
**ENERGY DISSIPATING SYSTEM COMPONENTS**				
At3g22370	Alternative oxidase 1a, AOX1A	DVNHFASDIHYQGR	329	342
		GIASYWGVEPNK	103	114
		GNIENVPAPAIAIDYWR	290	306
		LPADATLR	307	314
At5g64210	Alternative oxidase 2, AOX2	IENVAAPAIAIDYWR	291	305
		VVGYLEEEAIHSYTEFLK	267	284
		DVNHFASDIR	328	337
		IPTDIFFQR	164	172
**MITOCHONDRIAL MEMBRANE BIOGENESIS**				
At4g04870	Cardiolipin synthase (CMP-forming), CLS	DLLHPGLVGIVLLR	222	235
		DVALVGGAVYLR	236	247
		LLQSATPLHWR	73	83
		VEPLFISK	272	279
**CHAPERONE AND MEMBRANE SCAFFOLD PROTEIN**				
At5g40770	Prohibitin 3, PHB3	TKPHTFSSISGTK	76	88
		EIASTLAR	251	258
		VLSRPEVSR	99	107
		AVIFDR	41	46
**TRANSPORT PROTEINS**				
At3g01280	Mitochondrial outer membrane protein porin 1, VDAC1	FSITTFSPAGVAITSTGTK	29	47
		DSTITVGTQHSLDPLTSVK	210	228
		SFFTISGEVDTK	249	260
		EDLIASLTVNDK	167	178
At5g15090	Mitochondrial outer membrane protein porin 3, VDAC3	GSLFLGDVATQVK	49	61
		DDLTASLILNDK	167	178
		SFFTVSGEVDSK	247	258
		HFNAGFNFTK	157	166

AGI, Arabidopsis Genome Initiative Identifier (TAIR); Protein, protein name; Sequence, peptide sequence; Sequence localization, localization of the peptide within the protein sequence.

* According to UniProt;

** According to TAIR; “marked in red color”—Peptides detected by MRM;

# *SIS could not be detected by MRM*.

#### Isotopically labeled standard peptides

Stable-isotope labeled standard (SIS) peptides, synthesized using isotopically labeled amino acids on the C-terminus: Arg ^13^C_6_; ^15^N_4_ (98% isotopic enrichment) or Lys ^13^C_6_; ^15^N_2_ (98% isotopic enrichment) were purchased from JPT Peptide Technologies GmbH (Berlin, Germany) as SpikeTides_L in ~20 nmol amounts made using the proprietary SPOT peptide synthesis technology.

#### Nano-LC-MRM optimization and analysis

MRM analysis was performed using a Waters Xevo TQ mass spectrometer (Waters, MA, USA) coupled to a Waters nanoAcquity Ultra Performance Liquid Chromatography (UPLC). Mobile phase A was 0.1% formic acid (FA) in liquid chromatography-mass spectrometry (LC-MS) grade water, and mobile phase B was LC-MS grade acetonitrile (ACN) with 0.1% FA. Peptides were loaded onto a Waters 2G nanoAcquity UPLC Symmetry C18 trap-column (180 μm × 20 mm, 5 μm particle size) and separated using a 60 min LC run, with a gradient of mobile phase B changing from 1 to 10% from 0 to 10 min and from 10 to 50% from 10 to 40 min on a Waters nanoAcquity UPLC BEH130 C18 Column (75 μm × 150 mm, 1.7 μm particle size). Other MS instrument parameters included: capillary voltage of 3.5 kV, purge gas flow of 100 L/h, cone gas flow of 5 l/h, NanoFlow gas set at 2.0 Bar, and source temperature of 150°C.

MRM parameters were empirically optimized for highest sensitivity using pure SIS peptides by selecting optimal precursor ions and most intense fragment ions, and determining their optimal cone voltage (CV) and collision energies (CE), respectively. The procedure was as described by Bakun et al. (2012), with the addition of testing the 20 most likely b-and y-series fragment ions based on the Global Proteome Machine *A. thaliana* spectral library database (5/30/13) observations when possible otherwise using the Peptide Tandem Mass Spectrum Predictor tool (Arnold et al., 2006). Top five transitions that produced the strongest signals, with their optimized CV and CE parameters, were selected and checked for signal interferences when present in buffer and a sample-digest background. MRM scans were conducted with the optimized settings at unit resolution, five transitions per peptide, and with dwell times of 10 ms for each transition. Data was analyzed using the Skyline Ver. 2.5.0 software (University of Washington, MacCoss Lab, Department of Genome Sciences, UW). The abundance of the natural peptide target was reported as the Natural to Heavy Ratio, which is the sum of the peak areas of all transitions for the natural target divided by the sum of the peak areas of transitions for the corresponding heavy standard peptide. All integrated peaks were manually inspected to ensure correct peak detection and accurate integration. All peptides were quantitated using 3 to 5 MRM interference free ion pairs per peptide. Gel fractions that contained detectable peptide signals were summed equally across the samples analysis. Statistical significance between *ftsh4* mutant and wild-type seeds was performed using a two-tailed Student's *t*-test.

### SDS-PAGE and western blotting

Total seed protein extracts (40 μg) were resolved on SDS-PAGE using 12% gels and transferred onto polyvinylidene difluoride (PVDF) or nitrocellulose membrane (Bio-Rad). Immunoblots were then probed with the appropriate primary mitochondrial antibodies (Table S1) and anti-rabbit secondary antibodies conjugated to horseradish peroxidase (Agrisera). The proteins of interest were visualized by chemiluminescence with the WesternBright Quantum Western Blotting Detection Kit (Advansta) and the GBox imager (Syngene). The immunoblot band intensities were quantitated using ImageJ Fiji software (Fiji). At least three biological replicates of each type of seeds were analyzed. Statistical significance between *ftsh4* mutant and wild-type seeds was performed using a two-tailed Student's *t*-test.

### RNA isolation and quantitative real-time PCR analysis

Total RNA from dry and germinating wild-type and *ftsh4* seeds (see chapter 2.3) was obtained using the isolation method consisting of a three-step extraction with organic solvents and precipitation with isopropanol and lithium chloride (Suzuki et al., 2004, modified). Seeds were ground in liquid nitrogen and the seed powder was mixed with 5 volumes of the extraction buffer (0.4 M LiCl, 100 mM Tris, pH 8.0, 25 mM EDTA, pH 8.0, 1% SDS) followed by adding of 5 volumes of chloroform:isoamylalcohol (24:1). The homogenate was shaked vigorously for 10 secs and centrifuged at 12,000 × g, 4°C, for 3 min. The upper phase was mixed with 1 volume of chloroform:isoamylalcohol (24:1) and centrifuged as above. The upper phase was then mixed with 1 volume of the phenol mixture and chloroform:isoamylalcohol (24:1) at a 1/2 volume of the phenol mixture, shaked vigorously for 10 sec and centrifuged at 12,000 × g, 4°C, for 3 min. The upper phase was mixed with 1/3 volume of 8 M LiCl and incubated for 60 min at −20°C. The mixture was centrifuged at 12,000 × g, 4°C, for 30 min. The obtained pellet was dissolved in RNase-free water and mixed with 1/10 volume of 3 M sodium acetate (pH 5.2) and 1 volume of isopropanol. The mixture was incubated for 10 min at room temperature and centrifuged at 12,000 × g, 4°C, for 15 min. The RNA was washed with 75% ethanol, air-dried and dissolved in a small amount of RNase-free water. The cDNA synthesis reaction was carried out using up to 2 μg of total RNA and a reverse transcription kit (Applied Biosystems). Quantitative real-time PCR analyses (qRT-PCR) were performed using the LightCycler 4.0 instrument (Roche Applied Science) and real-time 2x PCR Master Mix SYBR version B (A&A Biotechnology). The wild-type plant seeds served as a calibrator and the *HBT* gene (At2g20000) was used as a reference (Graeber et al., 2011). At least three biological replicates obtained from every studied time point were analyzed. All primers used for qRT-PCR are listed in Table S2.

### Seed respiration measurements

Oxygen uptake by intact wild-type and *ftsh4* seeds was measured in 50 mM HEPES, pH 7.2, at 30°C, using a Clark-type O_2_ electrode (Oxytherm, Hansatech Ltd, Norfolk, UK). Ten to twenty micrograms of dry seeds were stratified for 48 h (4°C, in the dark), then either directly transferred to the chamber for O_2_ consumption measurements or germinated for a given time at 30°C followed by measurements of oxygen uptake. To estimate the cytochrome *c* oxidase (COX)-mediated or alternative oxidase (AOX)-mediated O_2_ consumption by germinating seeds the measurements were performed in the presence of 3 mM salicylhydroxamic acid (SHAM) or 2 mM potassium cyanide (KCN), respectively. Statistical significance between *ftsh4* mutant and wild-type seeds was performed using a two-tailed Student's *t*-test.

## Results

### Delayed germination of *ftsh4* mutants

The transgenic lines *ftsh4-1* and *ftsh4-2* lacking the FTSH4 protease were previously characterized by Gibala et al. (2009) who showed that *ftsh4-1* mutant seeds displayed a slower germination rate under both a long-day photoperiod (LD, 16-h light/8-h dark) and a short-day photoperiod (SD, 8-h light/16-h dark) at 22°C, when compared with those of wild-type seeds.

**Figure 2:**
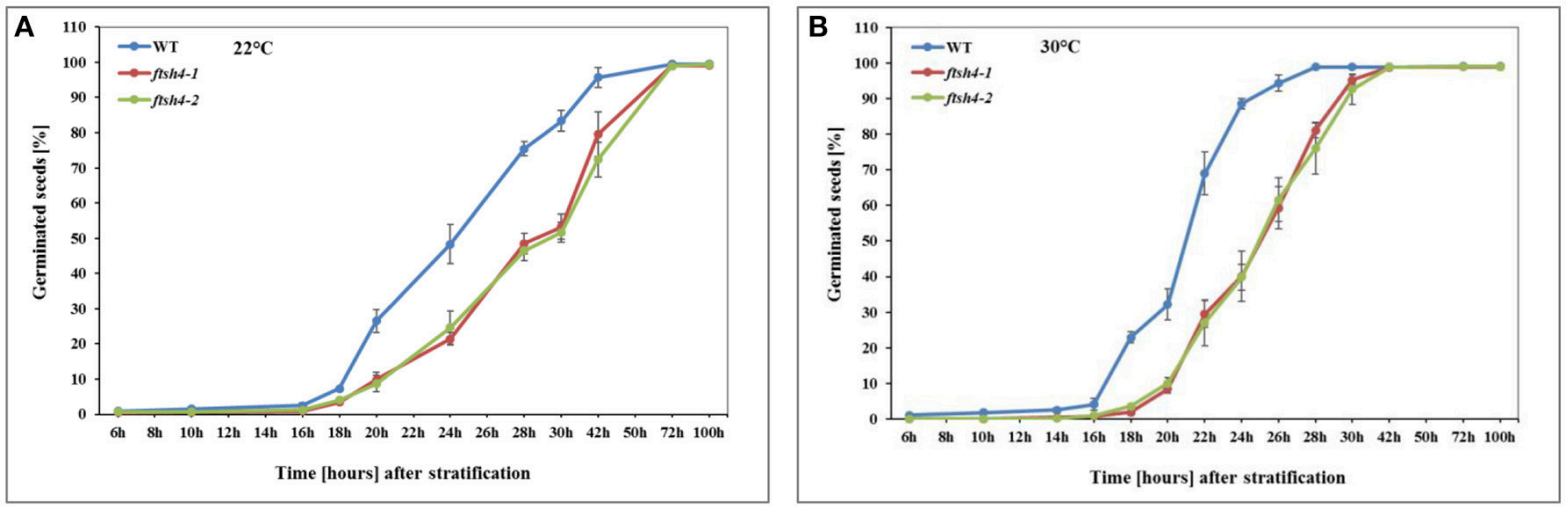
Germination rate of wild-type (WT) and *ftsh4* mutant seeds under long-day photoperiod and 22°C **(A)** and 30°C **(B)**. In all experiments, seeds were sowed on a black nitrocellulose membrane wetted with sterile distilled water, stratified for 48 h at 4°C in the dark and then incubated under 16-h light and 8-h dark (long-day photoperiod, LD) conditions at 22°C or 30°C. Seeds were scored every several hours until germination rate reached over 98%. A seed was considered as germinated after the emergence of the radical tip through the seed coat. **(A)** Germination of WT and *ftsh4-1* and *ftsh4-2* mutants under LD, 22°C. Data are mean ± SD of four independent biological replicates (*n* = 4, 140–180 seeds of each type). **(B)** Germination of WT and *ftsh4-1* and *ftsh4-2* mutants under LD, 30°C. Data are mean ± SD of five independent biological replicates (*n* = 5, 100–200 seeds of each type).

In this work, we performed germination assays of WT and *ftsh4-1* and *ftsh4-2* mutant seeds under optimal (LD, 22°C) and a moderately elevated (LD, 30°C) temperature (Figure 2, Figure S2). Before the actual germination assays, we also treated the seeds with moist chilling in darkness (stratification). The representative germination curve from 22°C (Figure 2A) shows that in 50% of WT seeds the radicle emerged after ~24 h, while in *ftsh4-1* and *ftsh4-2* mutant seeds 29 h after shifting the seeds from stratification conditions to 22°C. A similar delay in germination (~4–5 h) between *ftsh4* mutant seeds and WT has been observed when the seeds were incubated under a slightly higher temperature (30°C) (Figure 2B). As the difference in germination kinetics between WT and the mutant is similar at both temperatures, but the germination is completed faster at 30°C, all further experiments were conducted at slightly higher temperature. As displayed in Figures S2A,B, almost all WT seeds (based on radicle extrusion) were determined as being fully germinated after ~24–28 h of germination at 30°C, but the *ftsh4* mutant seeds were considered as fully germinated only after ~38–42 h of germination (Figure 2B, Figure S2B).

### Expression of the FTSH4 protease during germination

**Figure 3:**
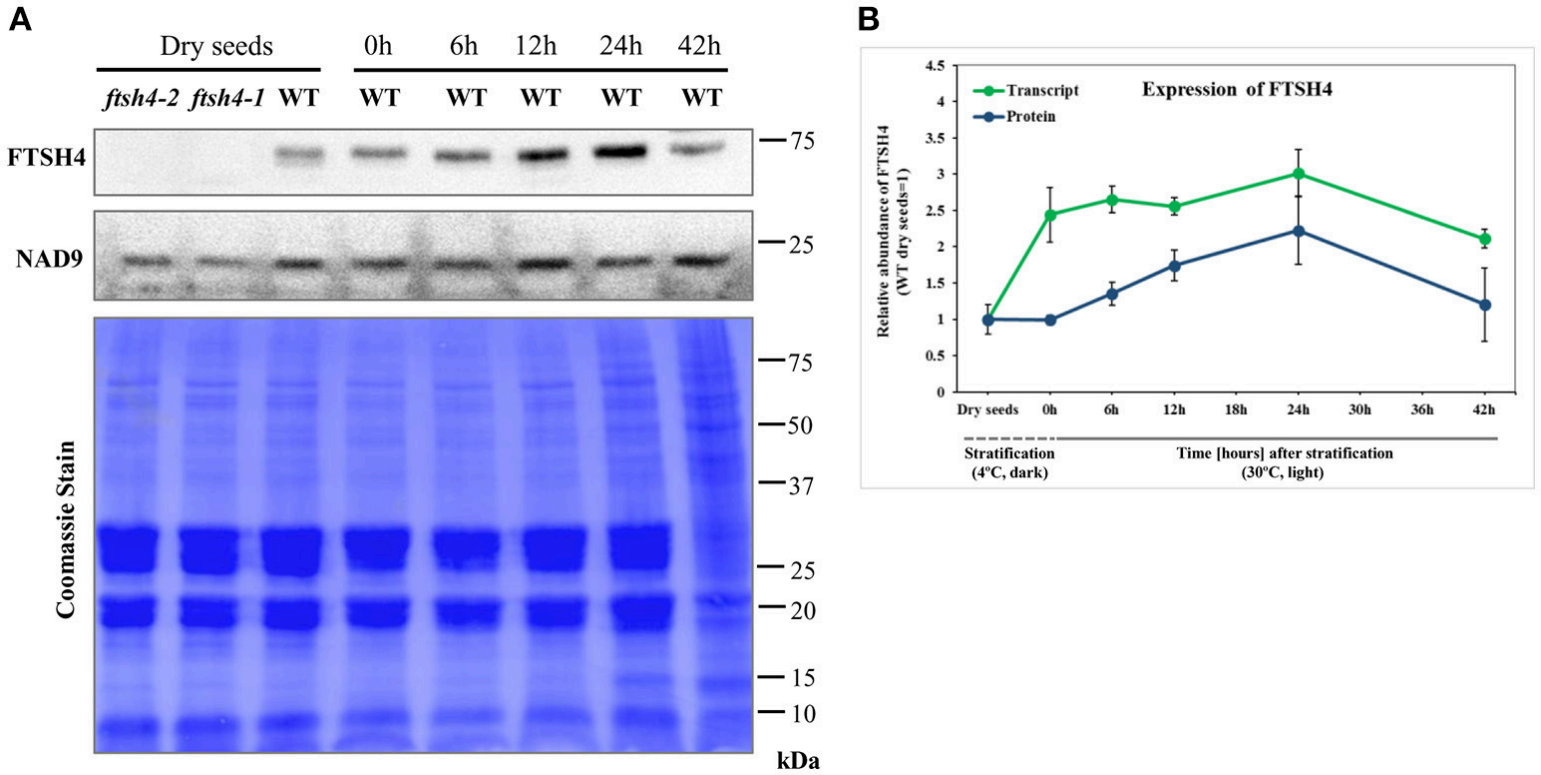
Western blot analysis of FTSH4 abundance in Arabidopsis seeds. **(A)** Total seed protein extract has been obtained from wild-type (Col-0) dry seeds, seeds that were stratified at 4°C for 48 h and not germinated (0 h) as well as seeds which after stratification were subjected to germination under long-day photoperiod (LD) and 30°C for 6, 12, 24, and 42 h. The samples were separated by SDS-PAGE (12%), transferred onto a membrane and subjected to immunodetection with antibodies anti-FTSH4. NAD9 protein levels are shown as a control. Membrane stained with Coomassie Brilliant Blue (CBB) is presented to show an equal loading. **(B)** Quantification of FTSH4 abundance at the level of transcript (in green) and protein (in blue) in dry, stratified and germinating at 30°C wild-type seeds. Transcript abundance was quantified with qRT-PCR. The values are given as relative to the value obtained for dry seeds (set as 1). Protein amount was quantified densitometrically using ImageJ (Fiji) based on immunodetection with antibodies directed against FTSH4 as well as Ponceau S or CBB (Coomassie Brilliant Blue) staining of the membrane as a loading control. The values are given as relative to the value obtained for dry seeds (set as 1). Mean values ± SD from at least three independent experiments are shown.

To monitor the expression of FTSH4 during germination, we examined the level of transcript and protein abundance of this protease in dry seeds, stratified seeds (0 h) and seeds collected after 6, 12, 24, and 42 h of germination at 30°C by quantitative PCR analysis and immunodetection, respectively (Figure 3). Both analysis showed that the FTSH4 protease is present in dry seeds and its level gradually increases after seed stratification, peaking after 24 h when the process of germination *sensu stricto* ends, and subsequently decreases. Thus, the dynamic changes of FTSH4 protein reflect changes in *FTSH4* transcript levels throughout the germination course. Taken together, a high abundance of FTSH4 protein and transcript in dry and germinating seeds as well as defective germination of *ftsh4* mutants, strongly support the importance of the FTSH4 protease for seed germination.

### Defining the protein targets and optimization of MRM

To attain a deeper knowledge about the involvement of FTSH4 in the process of mitochondrial biogenesis during germination, we applied the targeted proteomics approach, MRM, to quantify the abundance of certain mitochondrial proteins in Arabidopsis wild-type and *ftsh4-1* and *ftsh4-2* seeds. Because the properties of Arabidopsis seeds make an isolation of the mitochondrial fraction practically impossible, the MRM measurements were performed using a total seed protein extract, which was prepared from dry mature seeds (DS), stratified seeds (0 h), and from seeds collected at a different time course of germination (6, 12, 24, and 42 h) under slightly elevated temperature (30°C).

Key steps in the MRM design and workflow for quantification of *A. thaliana* mitochondrial proteins in total seed protein extract are summarized in Figure 1. First, we generated a list of mitochondrial protein targets mainly based on our previous experimental data concerning the *ftsh4* mutants (Kolodziejczak et al., 2007; Smakowska et al., 2016) and publications on seed-specific proteins (Elorza et al., 2006; Macherel et al., 2007; Restovic et al., 2017) (Table 1). These protein targets belong to the components of the OXPHOS (subunits of complexes I, II, III, IV, and ATP synthase) and are encoded by nuclear (NAD75, CA2, SDH1-1, SDH2-2, RIESKE, COX5C-1, ATP2-1) or mitochondrial DNA (NAD7, COB, COX2, ATP1). For the MRM study we also selected AOX1A, proteins that are involved in transport (VDAC1 and VDAC3), inner membrane scaffold (PHB3) as well as proteins highly specific to dry and germinating seeds, such as AOX2, SDH2-3, and CLS (Macherel et al., 2007). Next, a total of 71 specific peptides, whose sequences are unique to each selected mitochondrial target protein, and which have often been or are likely to be observed in MS experiments, were selected (Table 1). To increase the robustness of the MRM analysis and to protect against false positive quantitation, we employed stable isotope-labeled standard peptides (SIS) and used these as internal standards for each selected target peptide. These standards, containing an identical amino acid sequence to the target peptide, co-elute and produce identical fragment spectra in LC-MS analysis, greatly increasing the assay's specificity. Furthermore, the addition of SIS peptides in equivalent amounts to each sample allows for precise relative quantitation and normalization of a peptide amount across the analyses. The MRM method was additionally empirically optimized for highest sensitivity using pure SIS peptides. This increased the target peptide signal strength and therefore improved the sensitivity of detection, which is important for low abundance proteins. More detailed information is given in the Materials and Methods section.

### General view of MRM analysis of mitochondrial protein abundance in arabidopsis seeds

To examine the abundance of mitochondrial proteins in WT and *ftsh4* seeds by MRM we used a total seed protein extract, which was prepared from dry mature seeds, stratified seeds, and from seeds collected at a different time course of germination (6, 12, 24, and 42 h) at 30°C. We first studied total seed proteins precipitated with trichloroacetic acid/acetone, digested with trypsin and analyzed using the optimized MRM method. Unexpectedly, out of 71 specific peptides only 6 peptides of five proteins (ATP1, ATP2-1, VDAC1, VDAC3, PHB3) were observed with a good signal (above 10,000 counts intensity), while 18 peptides of 10 proteins (NAD7, CA2, SDH1-1, SDH2-3, COX2, COX5C-1, ATP1, ATP2-1, VDAC1, and PHB3) were detected with a low signal (below 10,000 counts intensity) (data not shown). The limited success of this approach indicated that further improvement of the sample preparation was necessary. We then employed a fractionation method based on separation of seed proteins on 12% polyacrylamide gel electrophoresis to enrich protein levels in the analyzed sample. Gel lines representing each studied sample (DS, 0, 6, 12, 24, and 42 h of WT, *ftsh4-1* and *ftsh4-2*) run as three independent biological replicates were manually excised and divided into 12 equivalent slices, which were then digested separately with trypsin and analyzed using the MRM method (Figure 1B, Data Sheets S1–S6). With the use of fractionation, additionally now for NAD7, NAD75, CA2, SDH1-1, SDH2-3, RIESKE, COX2, and COX5C-1, at least one peptide was observed with a good signal.

**Figure 4:**
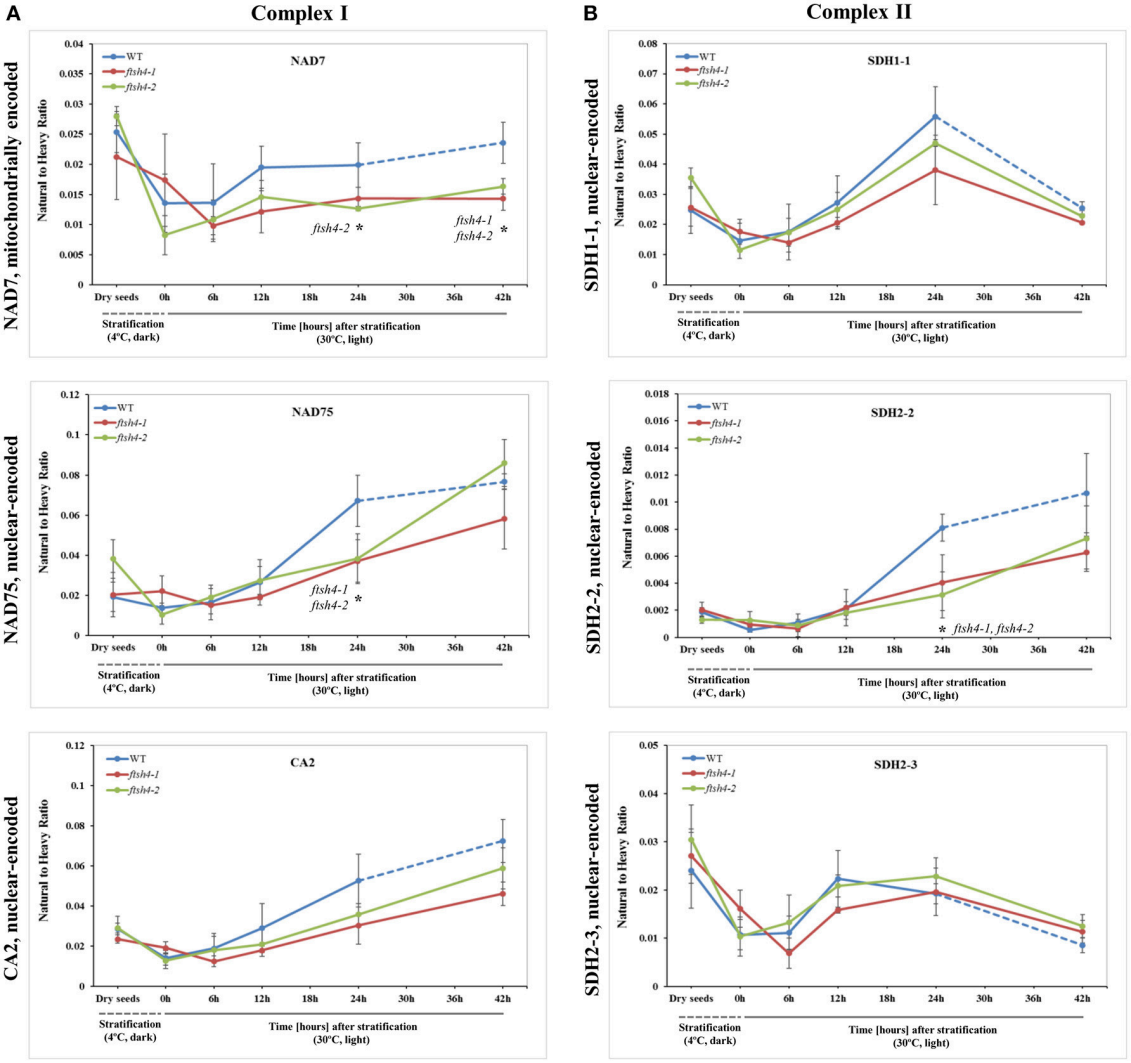
Quantitative time-course profiling of selected complex I **(A)** and complex II **(B)** subunits in *ftsh4* mutant seeds in comparison to those of WT during germination estimated by MRM. **(A)** Data show the abundance of NAD7 (AtMg00510) determined as the geometric mean of the peptides LVDIGTVTAQQAK, DIDSFTQQFASR and LLEFYER; NAD75 (At5g37510) and the peptides FASEVAGVQDLGILGR and NPAIIVGAGLFNR as well as CA2 (At1g47260) and the peptides NYINLAQIHASENSK, SFEQIEVER, and AIYTVGNWIR. **(B)** Data show the abundance of SDH1-1 (At5g66760) determined as the geometric mean of the peptides AFGGQSLDFGK, SSQTILATGGYGR and NSNGSLPTSTIR; SDH2-2 (At5g40650) and the peptide WNPDNPGKPELQDYK as well as SDH2-3 (At5g65165) and the peptides HLLSDPLVR, LQAITESETK and GLNPASAILK. The dashed line in the graph indicates the end of germination *sensu stricto* and the beginning of the next developmental phase. In all experiments, seeds were stratified and then incubated under LD conditions at 30°C. Stratification consisted of pre-treatment of seeds for 48 h at 4°C in the dark. Dry seeds, approximately 3-months-old dry seeds; 0 h, seeds after 48 h of stratification and no germination; 6, 12, 24, 42 h, seeds after 48 h stratification followed by germination for 6, 12, 24, and 42 h. Data are mean ± SD of three independent biological replicates. ^*^p ≤ 0.05.

**Figure 5:**
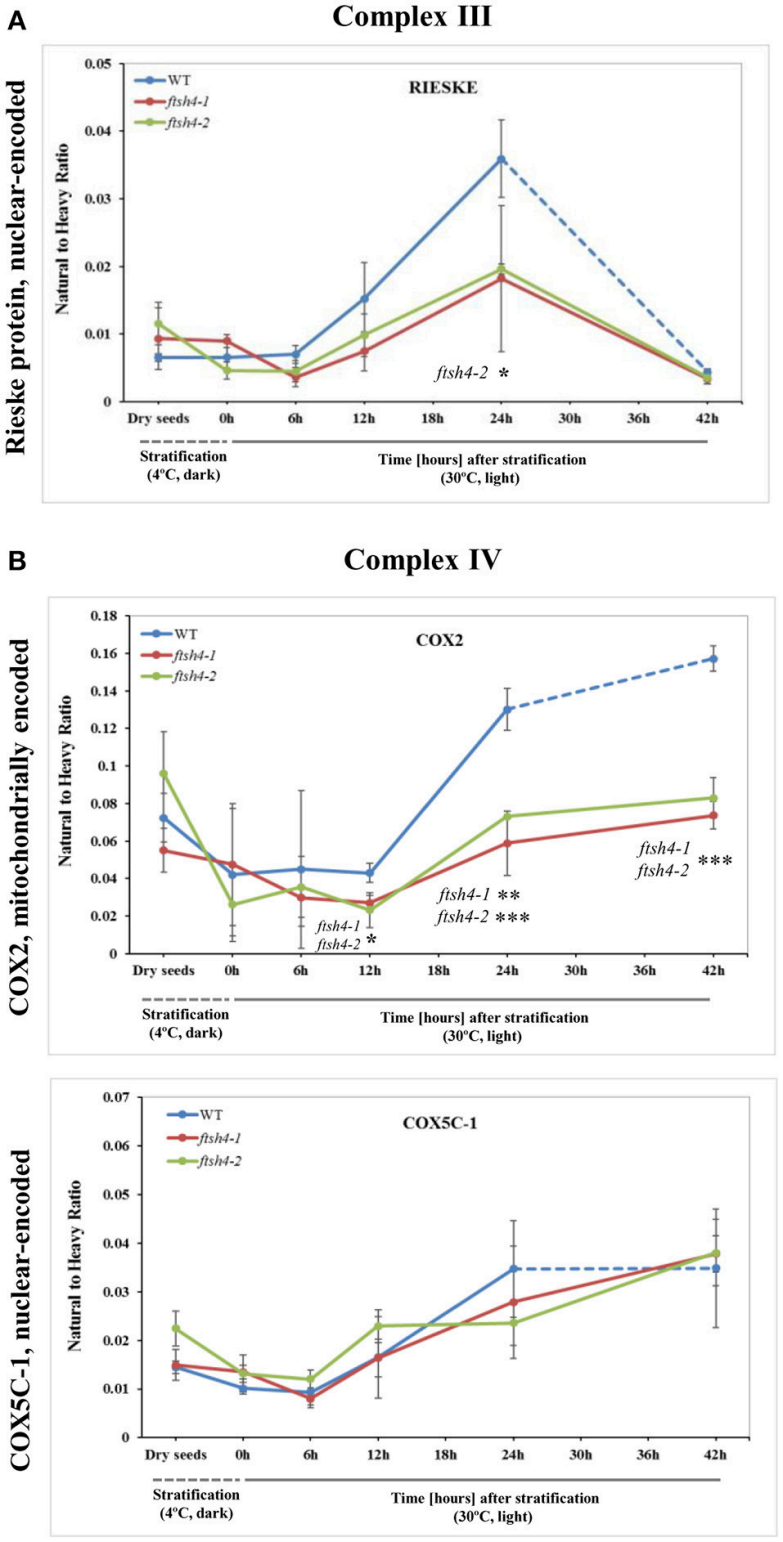
Quantitative time-course profiling of selected complex III **(A)** and complex IV **(B)** subunits in *ftsh4* mutant seeds in comparison to those of WT during germination estimated by MRM. **(A)** Data show the abundance of RIESKE protein (At5g13440) determined as the geometric mean of the peptides FVYASVLR and GPAPYNLEVPTYSFLEENK. **(B)** Data show the abundance of COX2 (AtMg00160) determined as the geometric mean of the peptides LLEVDNR, IIVTSADVLHSWAVPSLGVK and LNQISILVQR as well as COX5C-1 (At2g47380) and the peptide TFYDLLERCOX2. Other descriptions are the same as in Figure 4. Data are mean ± SD of three independent biological replicates. ^*^p ≤ 0.05; ^**^p ≤ 0.01; ^***^p ≤ 0.001.

**Figure 6:**
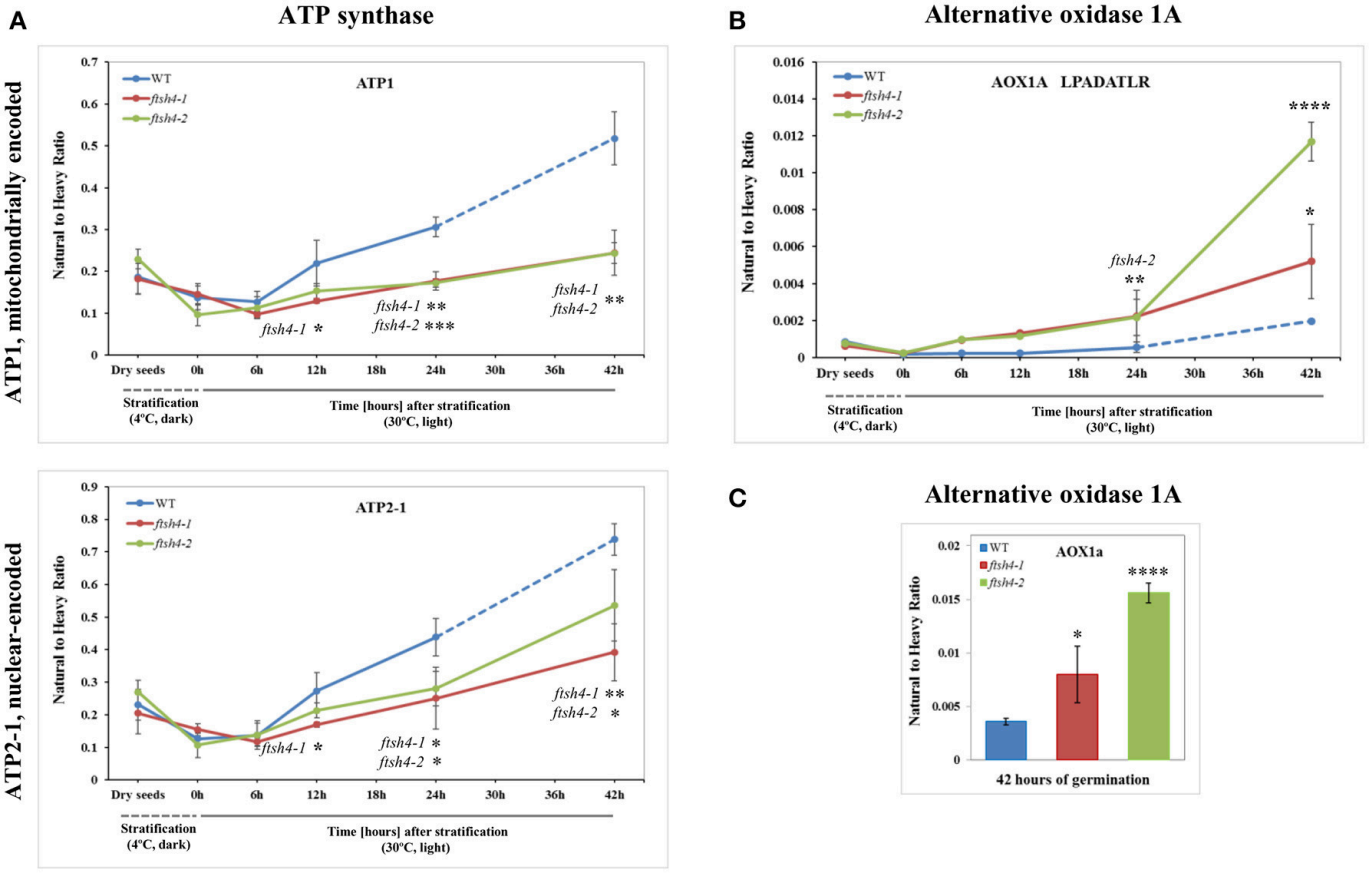
Quantitative time-course profiling of selected ATP synthase subunits **(A)** and alternative oxidase 1A (AOX1A) in *ftsh4* mutant seeds in comparison to those of WT during germination estimated by MRM. **(A)** Data show the abundance of ATP1 (AtMg01190) determined as the geometric mean of the peptides GIRPAINVGLSVSR, EAFPGDVFYLHSR, AVDSLVPIGR and TTIAIDTILNQK as well as ATP2-1 (At5g08670) and the peptides FTQANSEVSALLGR, DAPALVDLATGQEILATGIK and VGLTGLTVAEYFR. **(B)** Data show the abundance of AOX1A (At3g22370) using the peptide LPADATLR. **(C)** Data show the abundance of AOX1A determined as the geometric mean of the peptides GIASYWGVEPNK and DVNHFASDIHYQGR in seeds after 42 h of germination. Data are mean ± SD of three independent biological replicates, with the exception of AOX1A and peptide LPADATLR **(B)**, where the results of one replicate for dry seeds, 0, 6, and 12 h are shown. ^*^p ≤ 0.05; ^**^p ≤ 0.01; ^***^p ≤ 0.001; ^****^p ≤ 0.0001. Other descriptions are the same as in Figure 4.

**Figure 7:**
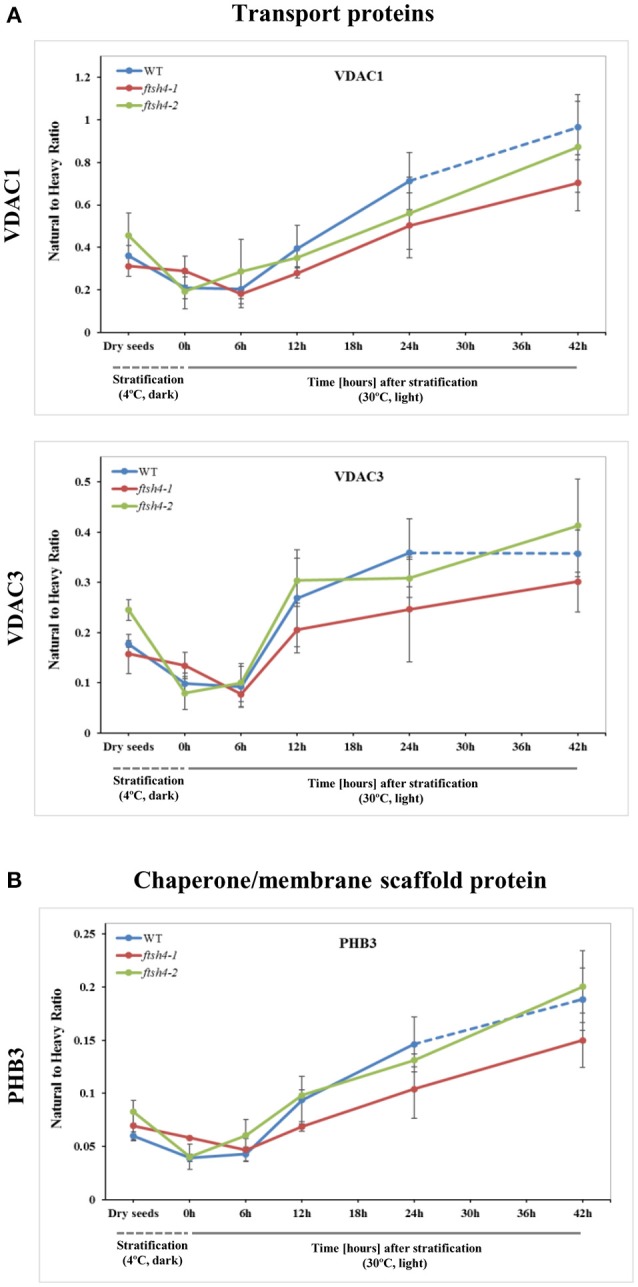
Quantitative time-course profiling of mitochondrial transport proteins, VDAC1 and VDAC3 **(A)**, and chaperone/membrane scaffold-like protein PHB3 **(B)** in *ftsh4* mutant seeds in comparison to those of WT during germination estimated by MRM. **(A)** Data show the abundance of VDAC1 (At3g01280) determined as the geometric mean of the peptides SFFTISGEVDTK, DSTITVGTQHSLDPLTSVK and FSITTFSPAGVAITSTGTK as well as the abundance of VDAC3 (At5g15090) and the peptides GSLFLGDVATQVK, SFFTVSGEVDSK and HFNAGFNFTK. **(B)** Data show the abundance of PHB3 (At5g40770) determined as the geometric mean of the peptides EIASTLAR, VLSRPEVSR and AVIFDR. Other descriptions are the same as in Figure 4. Data are mean ± SD of three independent biological replicates.

Taken together, using the seed protein fractionation by SDS-PAGE, we successfully quantified the abundance of 15 out of the 18 targeted mitochondrial proteins in dry seeds, and in each studied stage of the germination course of wild-type and *ftsh4* mutants detecting with high confidence 38 out of 71 selected peptides (Table 1, Figures 4–**7**, Figures S3–S7, Data Sheets S1–S6). Attempts to examine the abundance of AOX1A were partially ineffective. We could determine the abundance of AOX1A, detecting the LPADATLR peptide, in only one replicate of WT and *ftsh4* in the dry seeds, 0, 6, and 12 h samples, as well as in three replicates of WT and *ftsh4* seeds germinating for 24 and 42 h. Additionally, we detected the GIASYWGVEPNK and DVNHFASDIHYQGR targeted peptides of AOX1A in three replicates of WT and *ftsh4* seeds germinating for 42 h (Figure **6B**). SDH2-2 was likely in lower abundance as it was detected only with a single peptide with a low signal (Figure 4B). We were unable to reliably quantify the abundance of COB, AOX2, and CLS with any of the peptides due to an insufficient signal-to-noise ratio (data not shown).

In general, in this work more than one peptide was used to estimate the abundance of almost all the examined proteins. The amount of each measured protein is described as mean values (determined as the geometric mean) of all targeted peptides belonging to a particular protein (Figures 4–**7**). The single peptide-derived data is provided as Supplementary Information (Figures S3–S7, Data Sheets S1–S6). Most of detected peptides of an individual protein measured in either wild-type or mutant condition showed relatively similar kinetics in changes of abundance during germination.

### Differential abundance of selected mitochondrial proteins during the course of germination of wild-type and *ftsh4* seeds by MRM

**Figure 8:**
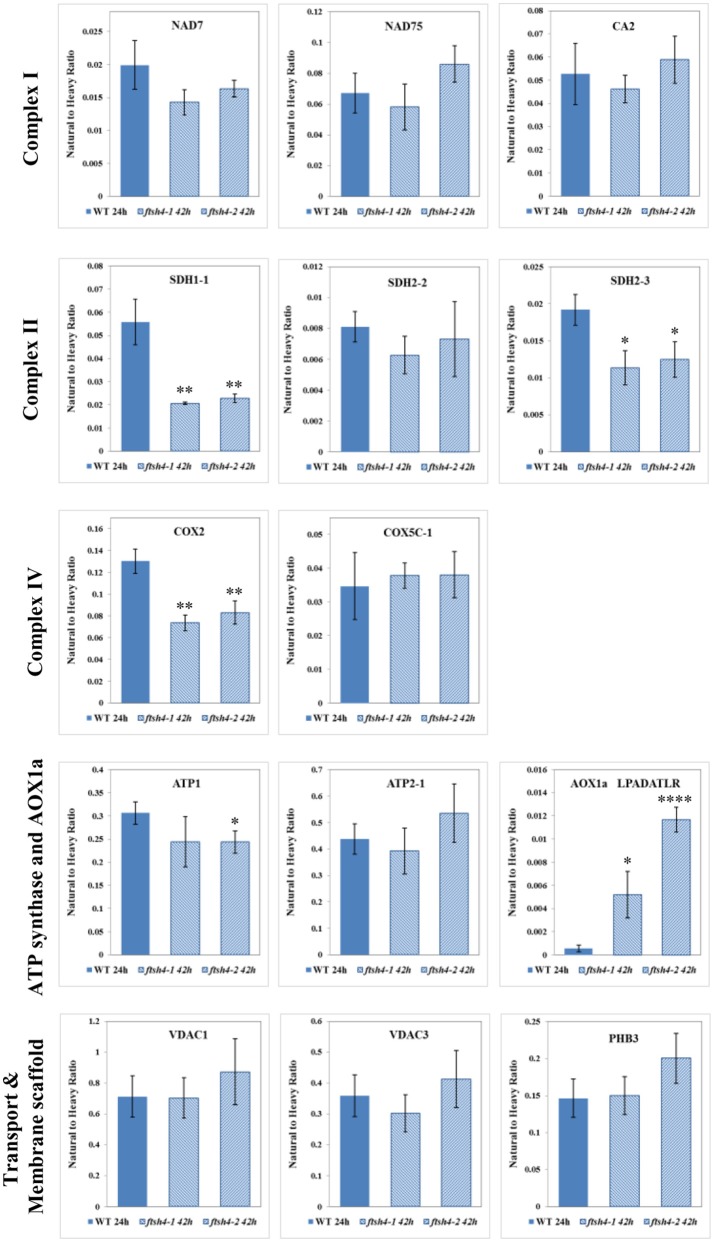
Comparison of the protein abundances between *ftsh4* seeds from the 42-h time point and wild-type seeds from the 24-h time point of germination. Protein abundance has been estimated by MRM. Details regarding the studied proteins and the selected peptides are given in Figures 4–7. Data are mean ± SD of three independent biological replicates. ^*^p ≤ 0.05; ^**^p ≤ 0.01; ^****^p ≤ 0.0001.

The abundance of NAD7, NAD75 and CA2, the subunits of complex I of the OXPHOS, increased steadily over the course of germination in both wild-type and *ftsh4* (Figure 4A, Figure S3), however, their abundance was slightly but not statistically significantly higher in WT than in the mutant §tarting approximately from the 6-h time point for NAD7 and 12-h time point for NAD75 and CA2. Notably, statistically significantly lower abundance was observed in the mutant after 24 and 42 h for NAD7 (~1.5-fold), and 24 h for NAD75 (~2-fold), when the same chronological age was considered. Furthermore, the level of NAD7 in dry seeds of both wild-type and mutants was substantially high, decreased dramatically during stratification (around 2-fold) and then increased gradually to a level lower than in dry seeds (Figure 4A, Figure S3). In contrast, a rather low abundance of NAD75 and CA2 was measured in WT and *ftsh4* dry seeds and during first 6 h after stratification, followed by a relatively strong increase (around 3-fold) in abundance over the time of germination. Notably, the observed decrease in abundance of the nuclear-encoded subunits of complex I in the *ftsh4* mutant (~2-fold for NAD75 and 1.5-fold but not statistically significant for CA2 at 24 h) in comparison to wild-type at the same chronological age was not so obvious as after comparing the similar developmental stages, when more than 90% of the seeds were considered as germinated. In *ftsh4* seeds after 42 h the abundance of NAD75 and CA2 was either almost equal or even slightly higher than that estimated in the wild-type after 24 h (Figure **8**). This tendency, however, was not found for the mitochondrially encoded NAD7, which showed a slightly but not statistically significantly lower level than in WT (Figure **8**).

To examine the changes in abundance of complex II in dry and germinating seeds, we measured the amount of the peptides for the flavoprotein subunit SDH1-1 and two iron-sulfur subunits: SDH2-2 and SDH2-3 (Figure 4B, Figure S4). In Arabidopsis, the *SDH2-2* subunit is expressed only in vegetative tissues while the expression of *SDH2-3* is fully seed-specific (Elorza et al., 2006). Regarding SDH2-2, we were able to detect only one peptide (WNPDNPGKPELQDYK) out of 4 targeted sequences (Table 1). The abundance of SDH1-1 and SDH2-2 in dry seeds and up to 12 h after stratification was very low and comparable between wild-type and *ftsh4*. After that time point, the abundance of both subunits displayed an increase up to 24-h time point, followed either by a further increase in abundance (SDH2-2) or a strong decline (SDH1-1) (~2-fold) after 42 h in both WT and *ftsh4* seeds (Figure 4B). In reference to the chronological age of WT and *ftsh4*, the abundance of SDH2-2 was significantly lower (around 2.6-fold) in the mutant than in the wild-type after 24 h of germination (Figure 4B). When the abundance of SDH1-1 and SDH2-2 was compared between *ftsh4* and wild-type at the similar developmental stage, that is after completion of germination *sensu stricto* (24 h for WT and 42 h for *ftsh4*), the abundance of SDH2-2 in *ftsh4* seeds was similar to that in WT, while the level of SDH1-1 was significantly lower (around 2.7-fold) than in wild-type seeds (Figure **8**).

The seed-specific SDH2-3 showed a distinct pattern of changes in the abundance compared to two other examined subunits of complex II, starting with a considerably high level of detected peptides in dry seeds, decreasing in abundance during stratification (~1.6-fold in WT and 3-fold in *ftsh4* seeds), and then increasing to reach the maximum at the 12 and 24-h time point of germination in wild-type and *ftsh4*, respectively. Afterwards, the abundance of SDH2-3 decreased continuously in both types of plants (Figure 4B). In contrast to the other examined OXPHOS subunits, the abundance of SDH2-3 was rather comparable between the mutant and wild-type during the course of germination, however, a slight difference in a shift in the peak of the subunit accumulation (after 12 h for WT and 24 h for *ftsh4*) was observed.

We were unable to reliably quantify any targeted peptides of COB, thus here the abundance profiling of complex III is determined only by the changes in the amount of RIESKE protein. Both detected RIESKE peptides displayed a similar profile in WT and *ftsh4* seeds, with an increase in abundance starting at the 6-h time point of germination, peaking after 24 h of germination, and rapidly declining (around 7-fold for WT and 3.6-fold for *ftsh4*) after 42 h of germination to a very low level in both WT and the mutant (Figure 5A, Figure S5A). In *ftsh4* seeds, however, the increase in abundance of RIESKE was less rapid, and after 24 h of germination its level in *ftsh4* constituted approximately half of that observed in wild-type (Figure 5A, Figure S5A). The dramatic decrease in the abundance of the RIESKE peptides indicates that active breakdown of this protein has occurred. This assumption is supported by the observation of additional MRM signals for the RIESKE peptide in the lower molecular weight fractions of the SDS gels in both wild-type and *ftsh4* mutants in all studied replicates of 42 h time-point (Data Sheet S6).

An examination of changes in the abundance of complex IV revealed a differential abundance pattern for mitochondrially encoded COX2, but not for nuclear-encoded COX5C-1, between wild-type and *ftsh4* seeds over the time of germination (Figure 5B, Figure S5B). The level of COX2 was low and relatively similar between WT and *ftsh4* up to 6 h of germination, but afterwards its abundance increased substantially in wild-type (~3.7-fold), while in *ftsh4-1* and *ftsh4-2* seeds this increase was significantly slower (around 2.3-fold). At the time when the *ftsh4* seeds were considered as fully germinated (42 h), the abundance of COX2 in the mutant was found at about half of that observed in WT seeds at the same developmental stage (24 h) (Figure **8**). In regard to COX5C-1, it was quantitated with a single peptide (TFYDLLER) as the additional two peptide standards could not be detected (Table 1). Unlike the aforementioned observations, no difference in neither abundance nor dynamics could be determined for COX5C-1 between WT and *ftsh4* mutant seeds (Figures 5B, **8**).

The abundance profiling of complex V was performed by measurement of the peptides unique to the ATP1 (mitochondrially encoded) and ATP2-1 (nuclear-encoded) subunits. We identified all four targeted peptides for ATP1 along with three out of four targeted sequences for ATP2-1 (Figure 6A, Figure S6A). We observed a progressive increase of ATP1 and ATP2-1 in both wild-type and *ftsh4* seeds over the time course of germination, starting roughly from the 6-h time point, however, the increase in the abundance of these subunits in the mutant was significantly slower than in the control seeds apparent soon after 12 h of germination (for ATP1 around 2.5-fold in WT and 1.7-fold in *ftsh4* seeds and for ATP2-1 around 3-fold in WT and 2-fold in *ftsh4* seeds between the time 12 and 42 h). After 42 h of germination of the mutant, when germination *sensu stricto* was completed, ATP1 was in slightly lesser amount than in wild-type seeds (statistically significantly lower in *ftsh4-2* but not in *ftsh4-1*) at the comparable developmental stage (24 h) (Figures 6A, Figures **8**). No difference in abundance was observed for ATP2-1, when two similar developmental stages were compared (Figure **8**).

Aside from the measurements of the abundance of the OXPHOS proteins, the MRM assay provided results for one of the targeted peptides (LPADATLR) of AOX1A for all the studied stages of WT and *ftsh4* germination (Figure 6B). We observed very slow but progressive accumulation of AOX1A in WT over the time course of germination, and after 24h, when germination *sensu stricto* was completed, the increase in the abundance of AOX1A became slightly faster. Notably, in *ftsh4* the marked increase in AOX1A abundance started already after stratification (6 h) and, at the end of germination (42 h), was ten (*ftsh4-1*) to more than twenty (*ftsh4-2*) times higher than in WT at the stage when almost all the seeds were considered as germinated (24 h) (Figures 6B, **8**). These results, however, should be interpreted with caution since only one biological replicate of the studied WT and *ftsh4* seeds from 0, 6, and 12-h time point resulted in reliable quantitative data. A markedly higher abundance of AOX1A in *ftsh4* seeds compared to WT has also been observed with two other targeted peptides, DVNHFASDIHYQGR and GIASYWGVEPNK (Figure 6C).

In the case of VDAC1, VDAC3, and PHB3, we did not find such noticeable differences in the abundance between WT and *ftsh4* during the time of germination as those observed for AOX1A and the majority of the OXPHOS subunits (Figure 7, Figure S7). Generally, all of these proteins showed a gradual increase in the abundance in both WT and *ftsh4* seeds and at the end of germination, namely after 24 h for WT and 42 h for *ftsh4*, their abundance in the mutant seeds was rather similar to wild-type (Figure 8).

### Abundance of selected mitochondrial proteins in germinating seeds of wild-type and *ftsh4* by western blotting

**Figure 9:**
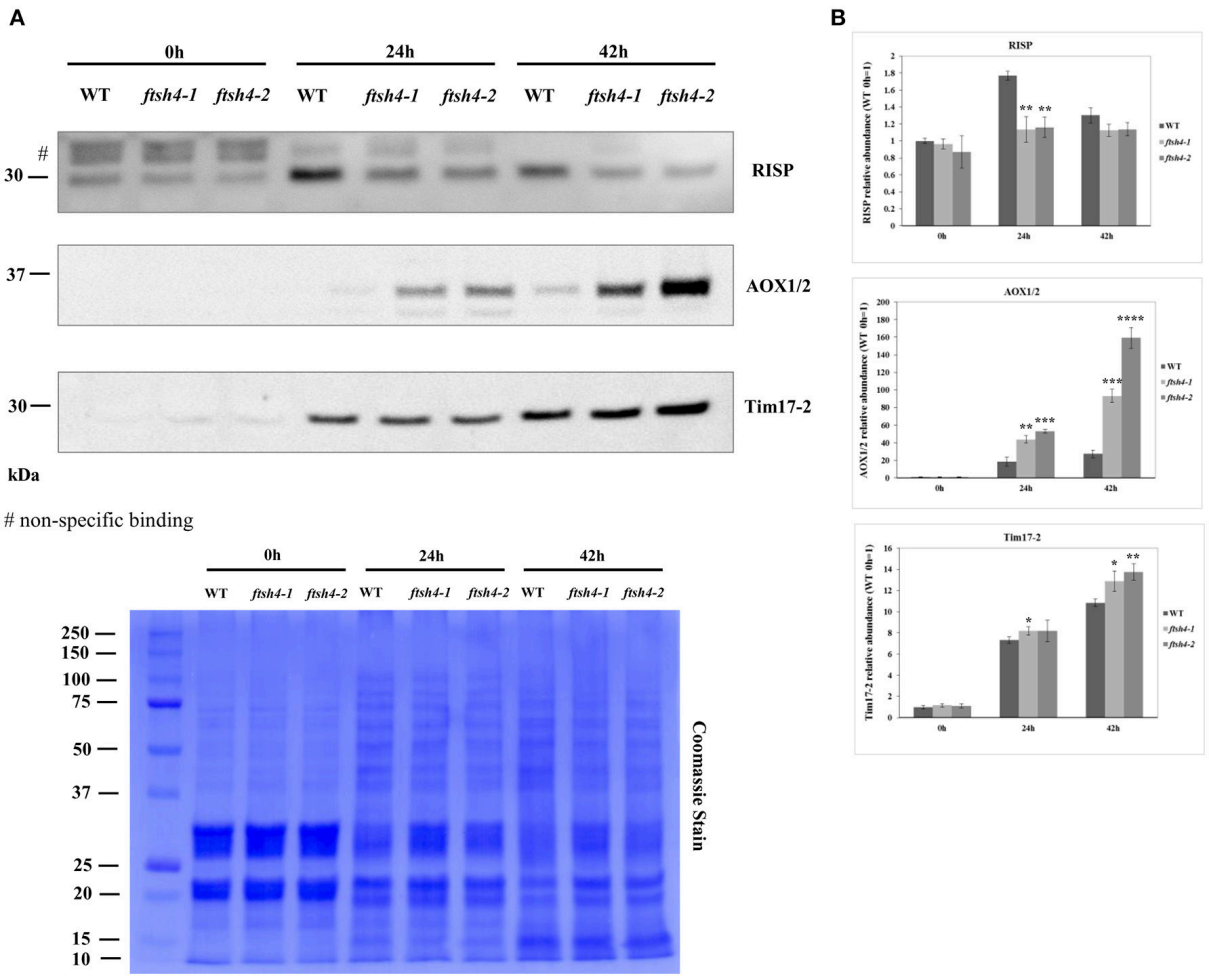
Western blot analysis of selected mitochondrial proteins in *ftsh4-1* and *ftsh4-2* mutant seeds in comparison to those of wild-type. In all experiments, seeds were stratified for 48 h at 4°C in the dark and then incubated for 24 and 42 h under LD conditions at 30°C. 40 μg of total seed protein extract was loaded on 12% SDS-PAGE. **(A)** Representative immunoblots with mitochondrial proteins detected after stratification (0 h) and after 24 and 42 h of germination at 30°C (24, 42 h). Representative membrane stained with Coomassie Brilliant Blue (CBB) is presented to show an equal loading. **(B)** Quantification of the abundance of the analyzed proteins. Protein amount was quantified densitometrically using ImageJ (Fiji) based on immunodetection with antibodies directed against selected proteins as well as Ponceau S or CBB staining of the membrane as a loading control. The abundance values are given as relative of the value obtained for WT 0 h seeds (set as 1). Data are mean ± SD of three independent biological replicates. ^*^p ≤ 0.05; ^**^p ≤ 0.01, ^***^ *p* ≤ 0.001; ^****^ *p* ≤ 0.0001.

In addition to the MRM analysis, in this work we performed protein quantification via western blotting of two respiratory chain proteins: RIESKE and AOX as well as an additional mitochondrial protein of interest, Tim17-2, in total seed protein extracts of WT and *ftsh4* mutants from stratified seeds (0 h) and seeds germinating for 24 and 42 h (Figure 9). We chose Tim17-2 in the light of our recent finding indicating that this protein, an essential component of the TIM17:23 translocase, is a proteolytic substrate of FTSH4 (Opalinska et al., 2017a). Because we could not include this protein within the MRM analysis anymore, we used western blotting.

The obtained results for RIESKE and AOX are consistent with the data presented by the MRM assay (Figures 5A, Figures 6B,C). Immunoanalysis showed that the level of Tim17-2 increased in both WT and *ftsh4* seeds during the time of germination, and slightly higher abundance of Tim17-2 was observed in the mutant when the samples were compared chronologically (Figure 9). Yet, the comparison of the stages when WT and *ftsh4* complete germination (24 h for WT and 42 h for *ftsh4*) and enter the seedling stage points out significantly greater (around 1.85-fold) amount of Tim17-2 in the mutant seeds than in wild-type.

### Transcript profiling of mitochondrial proteins quantified by MRM

**Figure 10:**
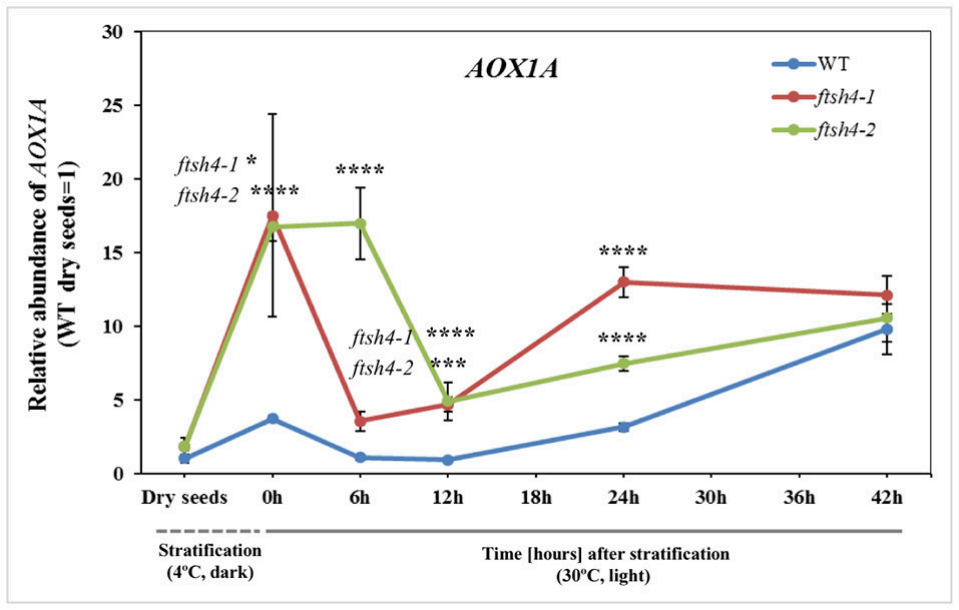
Abundance of AOX1A at the transcript level in dry, stratified and germinating wild-type and *ftsh4* seeds. Transcript abundance was quantified with qRT-PCR. The values are given as relative to the value obtained for WT dry seeds (set as 1). Data are mean ± SD of at least four independent biological replicates. ^*^p ≤ 0.05; ^***^p ≤ 0.001; ^****^p ≤ 0.0001.

To test whether the differential protein abundance in *ftsh4* seeds results from altered expression at the transcript level, we estimated with quantitative RT-PCR the abundance of transcripts for all proteins detected by MRM. In general, we did not observe distinct differences, meaning a transcript level more than two times higher/lower in both *ftsh4* mutant seeds, except for *AOX1A*, which displayed a several times higher abundance in *ftsh4-1* and *ftsh4-2*, compared to WT, in almost all studied stages (aside from dry seeds) (Figure 10, Figure S8). Interestingly, the rapid and significant increase in *AOX1A* abundance was observed in *ftsh4* seeds just after cold stratification and only slightly in the wild-type, and further again after ~12 h of germination (Figure 10). Some transcripts corresponding to OXPHOS such as *nad7, cox2* and *COX5C-1* exhibited in *ftsh4* seeds a slightly higher expression, with peak abundance after stratification (0 h) and declined thereafter (Figure S8). The seed-specific *SDH2-3* was also observed at a mildly elevated level in the mutant seeds after stratification (0 h) and during the early stages of germination (6, 12 h) and decreased afterwards. Similarly, in *ftsh4* seeds the level of *VDAC1* and *VDAC3* showed a somewhat stronger upregulation after cold stratification (0 h) and declined in the abundance during germination (Figure S8).

Overall, our results indicate that, with the exception of *AOX1A*, the differences in gene expression in dry, stratified, and germinating *ftsh4* mutants were fairly small and that the observed effects on protein abundance most likely occurred post-transcriptionally.

### Seed respiration assay

**Figure 11:**
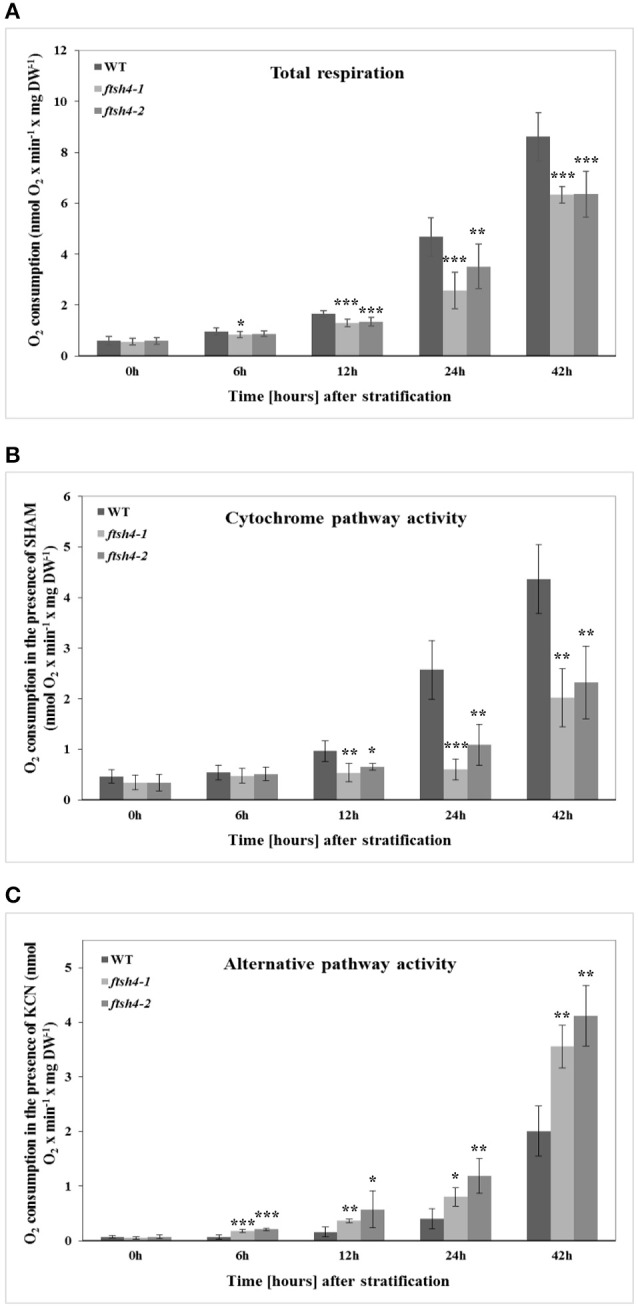
The time-course of respiration of intact WT and *ftsh4* seeds in the absence **(A)** or presence of either SHAM **(B)** or KCN **(C)**. Oxygen consumption rates (nmol O_2_ x min^−1^ x mg dry weight^−1^) by WT and *ftsh4* seeds were measured at 30°C using a Clark-type O_2_ electrode. **(A)** Total respiration of intact germinating WT, *ftsh4-1* and *ftsh4-2* seeds. **(B)** SHAM-insensitive respiration reflecting the COX-mediated O_2_ consumption by germinating WT and *ftsh4* seeds in the presence of 3 mM SHAM. **(C)** KCN-insensitive respiration reflecting the AOX-mediated O_2_ consumption by germinating WT and *ftsh4* seeds in the presence of 2 mM KCN. In all experiments, seeds were stratified and either directly transferred to the chamber for O_2_ consumption measurements (0 h) or germinated for a given time (6, 12, 24, 42 h) at 30°C followed by measurements of oxygen uptake. Stratification consisted of pre-treatment of seeds for 48 h at 4°C in the dark. Data are mean ± SD of at least four independent experiments. ^*^p ≤ 0.05, ^**^p ≤ 0.01, ^***^ *p* ≤ 0.001.

We performed measurements of oxygen consumption by intact wild-type and *ftsh4* seeds using a Clark-type electrode to determine whether the decrease in protein abundance of some of the OXPHOS components observed by the MRM assay in *ftsh4* results in a corresponding decrease in the activity of the cytochrome pathway. We first examined the total O_2_ uptake rate by stratified seeds (0 h) and germinating seeds of WT and *ftsh4* mutants. Regardless of seed type, we observed a progressive increase in the respiration rate over the time course of germination, however, the *ftsh4-1*, and *ftsh4-2* seeds exhibited a lower oxygen uptake rate compared to the control seeds (Figure 11A). A lower rate of respiration in the *ftsh4* seeds correlated with a significantly decreased activity of the cytochrome pathway in the mutant compared to wild-type estimated as SHAM-resistant respiration (Figure 11B). Notably, the cytochrome pathway activity in *ftsh4* after 42 h, when the seeds were considered as fully germinated, was slightly lower when compared to the activity of this pathway in WT at the comparable developmental stage (24 h). Thus, the pattern of the cytochrome pathway activity (Figure 11B) resembles that of the OXPHOS protein abundance profiles (Figures 4–6). In contrast, the total respiration of the mutant after germination *sensu stricto* (42 h) was definitely higher than the respiration of the wild-type measured at a similar stage (24 h). This was not surprising when the rate of the AOX-mediated oxygen consumption was estimated (Figure 11C). In agreement with the MRM assay indicating a great accumulation of AOX1A protein in *ftsh4*, the rate of O_2_ uptake in the presence of cyanide was much higher in the mutant than in wild-type. Altogether, the measurements of the total respiration rate as well as the activity of the COX- and AOX-mediated respiration in stratified and germinating seeds correlated well with the protein abundances observed by MRM and immunoblotting.

## Discussion

### The advantages and limitations of the MRM assay in mitochondrial protein studies in arabidopsis seeds

Among the proteomic methods used to quantify the abundance of mitochondrial proteins in seeds, there is relatively little information addressing quantitative studies of small-sized seeds such as those from *A. thaliana*. This is mostly because the fractionation of mitochondria from Arabidopsis seeds is practically impossible and thus the identification and quantitation of mitochondrial proteins must rely on the use of the whole seed protein extract. With the rapid advancement of mass spectrometry techniques, the application of targeted proteomics for the detection and quantitation of mitochondrial proteins in complex samples obtained from plant material has become a promising analytical tool in such studies (Hooper et al., 2017). In this work, we presented MRM as a sensitive and specific technique for estimating mitochondrial protein abundances in dry and germinating Arabidopsis seeds without the organelle enrichment. By using SDS-PAGE fractionation upfront in the sample preparation stage, and by optimizing MRM instrument settings using peptide standards (SIS) for an increased signal we successfully quantified the abundance of 15 out of the 18 targeted mitochondrial proteins in the complex total seed protein extracts of wild-type and *ftsh4*, detecting 38 out of 71 selected peptides. MRM analysis employing SIS is considered to be more specific than antibody-based methods and is now an established and proven analytical technique for reliable and precise protein quantitation (Picotti and Aebersold, 2012; Aebersold et al., 2013). Here, the addition of equivalent amounts of SIS peptides across the sample sets not only allowed for data normalization and reduced analytical variability, but it also helped to differentiate between protein isoforms of AOX, which western blotting analysis could not distinguish (e.g., AOX1A).

Besides the advantages, the targeted mitochondrial protein quantitation by MRM in Arabidopsis seeds showed some limitations. These presumably arose from the type of the studied tissue as well as the protein target itself. Difficulties in obtaining reliable peptide abundance data for AOX2, CLS, and COB suggest that these mitochondrial proteins are low-abundance proteins, which are poorly represented in the dry and germinating seed proteomes. This is surprising especially for AOX2 and CLS as the transcripts of these proteins have been shown to be specific to the mature and dry seed (Saish et al., 2001; Nakabayashi et al., 2005; Clifton et al., 2006; Macherel et al., 2007). To our knowledge, no data regarding the expression of AOX2 and CLS at the protein level in seed mitochondria has been provided so far. Recently, Fu et al. (2012) showed that in Arabidopsis leaves AOX2 is targeted to chloroplasts and substitutes for the plastid terminal oxidase. The lack of credible quantitation of AOX2, CLS and COB also indicates that there is a limit of detection for MRM in assessing individual organellar proteins in Arabidopsis whole seed extracts, and thus suggests a requirement for a better protein enrichment method than the one used in our study. Similar observations were made by Taylor et al. (2014) who were unable to quantify by MRM the less-abundant mitochondrial proteins, mMDH2, mACO1, and mACO2 in Arabidopsis leaf total protein extracts, but the measurements were successful using isolated mitochondria.

### Aberrant mitochondrial biogenesis in *ftsh4* seeds

Generally, the MRM analysis of nuclear- and mitochondrially encoded mitochondrial proteins of the OXPHOS as well as AOX1A, PHB3, and VDAC isoforms revealed that there were no significant differences in abundance between WT and *ftsh4* in dry seeds and right after stratification at 4°C (0 h). Usually a higher abundance of the studied proteins was observed in dry seeds than after stratification in both WT and *ftsh4* mutant. This is probably related to unavoidable damages of mitochondrial membranes during cold stratification, which severely affect seed metabolic activity (Yin et al., 2009). The highest difference in the protein amounts between the dry seed and 0 h stages was observed for SDH2-3 and NAD7 proteins.

After stratification, in WT and *ftsh4* seeds, the steady-state level of all studied mitochondrial proteins gradually increased over the time of germination reflecting biogenesis of mitochondria. However, when the amount of the OXPHOS proteins was analyzed chronologically in the *ftsh4* mutant compared to wild-type, a decrease was observed for most of the studied OXPHOS subunits in the mutant, starting usually between the 6 and 12-h time point after stratification (Figures 4–Figures 6). The exceptions were only subunits CA2, SDH1-1, COX5C-1, and seed-specific SDH2-3. Although the accumulation of nearly all the OXPHOS subunits in the mutant mitochondria was obviously slower throughout germination, the final abundance of these proteins after completion of germination *sensu stricto*, namely after ~24 h for WT and 42 h for *ftsh4*, was almost equal in the mutant in comparison to wild-type (Figure 8). In contrast, the mitochondrially encoded OXPHOS subunits displayed either highly lowered abundance (COX2) or slightly (but not significantly) decreased level (NAD7, ATP1) in the mutant seeds when comparing to WT after completion of germination (Figure 8). It should be underlined that the activity of the OXPHOS system in *ftsh4*, when the seeds were considered as fully germinated (42 h), was only slightly lower when compared to WT at the comparable developmental stage (24 h). Taken together, based on steady-state levels of OXPHOS subunits determined by MRM it is conceivable that a loss of FTSH4 delayed biogenesis of OXPHOS complexes and in consequence a longer period of time is required for the assembly of the oxidative phosphorylation system in the mitochondrial inner membrane. Moreover, in the mutant, OXPHOS complexes show deficiency of some subunits after completion of germination, however, this deficit affects the oxidative phosphorylation activity only slightly. Also, at this developmental stage a larger proportion of total respiration was attributed to the alternative pathway activity. Eventually, nearly all the mutant seeds completed germination.

**Figure 12:**
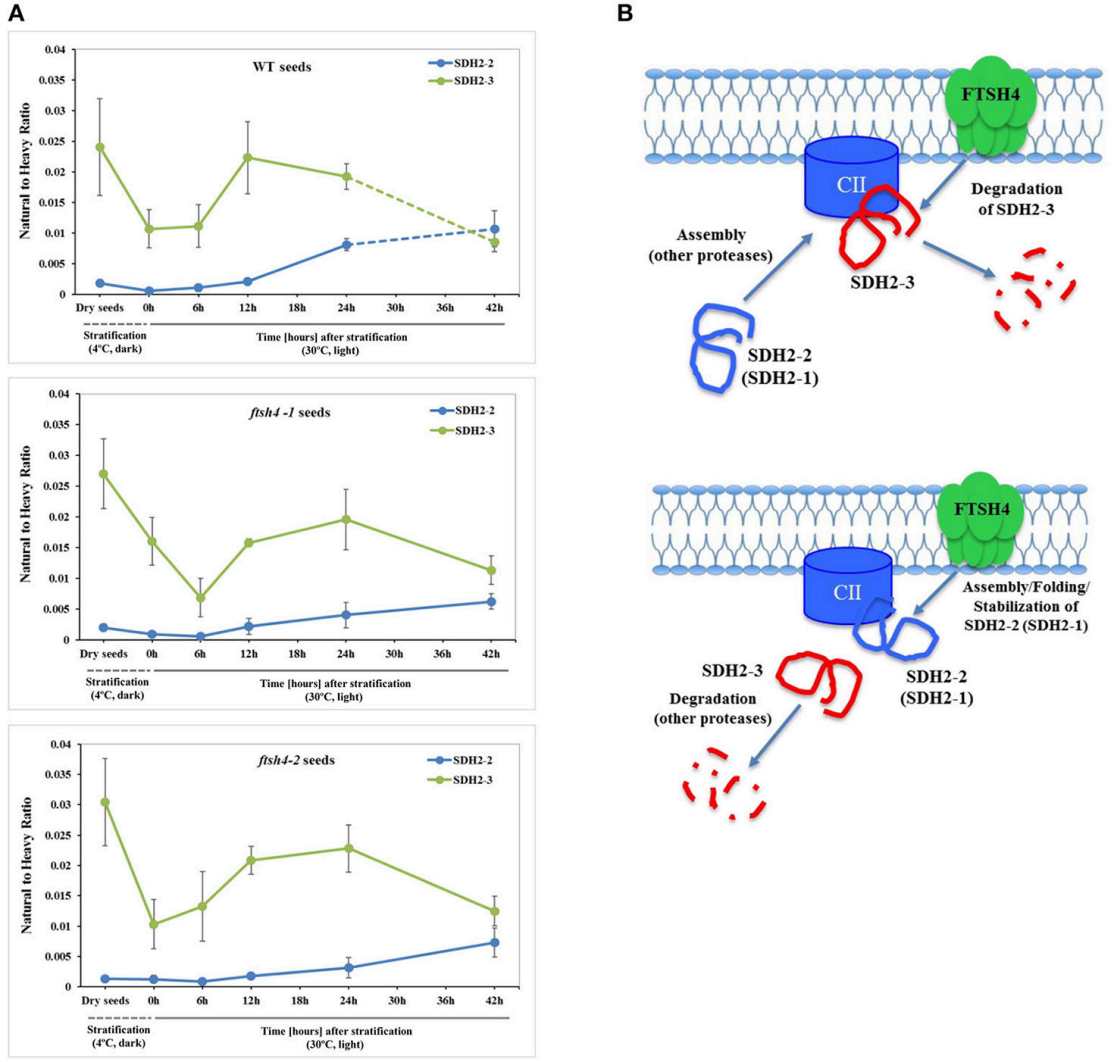
Biogenesis of complex II in germinating Arabidopsis seeds. **(A)** Quantitative comparative time-course profiling of the iron-sulfur subunits of complex II, SDH2-2 and seed-specific SDH2-3, in the wild-type and *ftsh4* seeds, estimated by MRM. Details regarding SDH2-2 and SDH2-3 abundance are given in Figure 4. The dashed line in the graph indicates the end of germination *sensu stricto* and the beginning of the next developmental phase. **(B)** Hypothetical role of the FTSH4 protease in the biogenesis of complex II during germination of *A. thaliana*. FTSH4 is involved in the degradation of the seed-specific SDH2-3 subunit of complex II at the end of the germination process (proteolytic function of the protease). FTSH4 is responsible for either the folding, assembly or stabilization of the SDH2-2 subunit of complex II (chaperone-like function of the protease).

One of the subunits which behaved unlike the majority of the examined OXPHOS subunits during germination of *ftsh4* seeds is the iron-sulfur subunit SDH2-3 of complex II. The abundance of this seed-specific subunit is almost comparable between WT and *ftsh4* throughout germination taking into account chronological age of plants and significantly lowered in *ftsh4* when two similar developmental stages were compared. It was reported based on the transcriptomic data that the expression of *SDH2-3* is fully seed-specific while the homologous SDH2-2 subunit is expressed only in vegetative tissues (Elorza et al., 2006). According to MRM data, the abundance of SDH2-3 in both WT and *ftsh4* was the highest in dry seeds followed by a substantial decline at the end of germination (Figures 4B, 12A). This strong decline in the abundance suggests that active breakdown of the SDH2-3 protein occurs. On the contrary, the amount of SDH2-2 was very low in dry seeds as well as in early germination stages and was gradually increasing over the time of germination. Our proteomic results strongly support earlier observations based on the RNA analysis (Elorza et al., 2006) and point out the exchange between SDH2-3 and SDH2-2 during biogenesis of complex II at the end of germination in Arabidopsis. It is of interest to note that a delay in exchange of these subunits was observed in the *ftsh4* mutant since the decrease in the abundance of SDH2-3 occurred in the mutant later than in wild-type (Figure 12A). It is tempting to speculate that the FTSH4 protease is involved in either the breakdown of SDH2-3 or the assembly of SDH2-2 into complex II and the loss of FTSH4 affected the time of the exchange of these subunits (Figure 12B).

Our MRM-based results imply the significance of the FTSH4 protease for the biogenesis of the OXPHOS in germinating Arabidopsis seeds. A similar conclusion about the function of FTSH4 was previously derived based on results coming from a combination of blue-native polyacrylamide gel electrophoresis and histochemical staining, however, at that time the results concerned other developmental stages of Arabidopsis (leaf mitochondria from older plants growing in SD or young seedlings growing under moderately elevated temperature) and were limited to assembly and activity of complexes I and V (Kolodziejczak et al., 2007; Smakowska et al., 2016). In this work, we not only showed the significance of FTSH4 for the biogenesis of complexes I and V in germinating seeds, but we additionally revealed for the first time the importance of this protease in the biogenesis of complexes II, III, and IV. Concerning complex III, in the *ftsh4* seeds we detected a decreased abundance of RIESKE, the iron-sulfur protein essential for maturation of this complex (Fernandez-Vizarra and Zeviani, 2017). It is noteworthy that a dramatic reduction in the abundance of the RIESKE peptides was observed in WT and *ftsh4* seeds at the last studied stage (42 h), however, the mechanism behind this process is unknown. Furthermore, significantly lower abundance of COX2, but not of COX5C-1 subunit of complex IV was found in *ftsh4* mutants. As COX2 is a central element of the catalytic core of complex IV (Soto et al., 2012), a strong reduction in the COX2 steady-state level implies that the assembly and stability of complex IV in *ftsh4* seeds is compromised.

Among the membrane-located non-OXPHOS proteins only AOX1A exhibited strong accumulation in *ftsh4* seeds, while VDAC1, VDAC3, and PHB3 showed no significant differences in abundance. Dual localization in the cell was reported for VDAC3 (Robert et al., 2012) and also recently for PHB3 (Seguel et al., 2018). Taking into account the fact that our MRM assay was conducted with the total seed protein extract, it is not possible to interpret the above-mentioned results in the aspect of the inner membrane biogenesis.

Additionally, we observed an earlier induction of the cytochrome pathway activity and complex IV abundance than AOX activity and level (more pronounced in wild-type but also in the mutant seeds) during germination. Given much lower affinity of AOX to oxygen as compared to complex IV (Millar et al., 1994), the observed difference probably reflects changes from highly oxygen-limiting to more aerobic conditions in germinating Arabidopsis seeds (Borisjuk and Rolletschek, 2009).

Furthermore, our results showed that the decrease in the abundance of the OXPHOS components in *ftsh4* germinating seeds was not associated with a transcriptional response but with alternations at the protein level. In contrast, soon after stratification and thus before an impairment in the OXPHOS in *ftsh4* seeds was evident (0 h), we observed a rapidly increased accumulation of AOX1A at the transcript level in the mutant seeds compared to wild-type. It is well established that expression of *AOX* genes is strongly induced at the transcript and protein level in response to dysfunction of the respiratory chain components (complexes I, III, and IV) and ATP synthase, cold or drought exposure or oxidative stress (Vanlerberghe and McIntosh, 1994, 1996; Karpova et al., 2002; Juszczuk et al., 2012). A dramatically higher transcript level of *AOX1A* in *ftsh4* seeds just after cold stratification suggests that during rehydration at 4°C the mutant seeds are under severe stress. This stress is rather not directly connected with *de novo* biogenesis of OXPHOS complexes since after stratification the abundance of all examined OXPHOS proteins has been the same in the mutant and wild-type. We recently documented altered content of membrane phospholipids in the *ftsh4* mitochondria (Smakowska et al., 2016). Given the fact that reorganization of membrane lipids is one of the major events occurring during seed imbibition/stratification (Yu et al., 2015), any perturbations in phospholipid content could lead to a defect in mitochondrial organization and, in consequence, induction of AOX1A transcription. Second induction of *AOX1A* expression in *ftsh4* seeds is probably related to a diminished biogenesis of the oxidative phosphorylation system. It corresponds with the time when a decreased abundance of the OXPHOS components and perturbation of the cytochrome pathway activity were apparent (Figures 10, 11).

The question arises how the loss of FTSH4 protease leads to a decreased abundance of most examined OXPHOS subunits. In other words, how does FTSH4 control the biogenesis of all the OXPHOS complexes at the post-transcriptional level? Given the dual nature of FTSH4 as a protease and a molecular chaperone with the data reported so far, several mechanisms that are not mutually exclusive could be considered. The preferred mechanism is based on the previous results pointing to FTSH4 being a part of a regulatory pathway that influences the abundance of some phospholipids including cardiolipin (CL) in plant mitochondria (Smakowska et al., 2016). The altered membrane phospholipid composition could delay the formation of OXPHOS complexes. Decreased abundances of individual complexes I and III_2_ as well as the supercomplex I_1_III_3_ were reported in mutants lacking CL (Petereit et al., 2017). The lower abundance of the OXPHOS subunits in the *ftsh4* mutant could also be linked to a role of FTSH4 as a chaperone aiding the proper folding of newly imported and/or synthesized proteins and assembling them into mature complexes. We did not find accumulation of any of the studied proteins in *ftsh4* seeds, however, we cannot exclude the possibility that an excess of unassembled OXPHOS subunits is degraded by other ATP-dependent mitochondrial proteases.

The next putative mechanism emphasizes also the chaperone-like activity of FTSH4, but in the context of translocations of OXPHOS proteins across the inner mitochondrial membrane and their delivery into the premature complex. The link between FTSH4 and the mitochondrial preprotein import machinery was recently documented (Opalinska et al., 2017a). The Tim17-2 protein, an essential component of the inner mitochondrial membrane translocase (TIM17:23) was recognized as proteolytic substrate of FTSH4. Immunological detection of Tim17-2 during germination revealed a significantly higher abundance of this protein in *ftsh4* at this stage, when the mutant seeds complete germination and enter the seedling stage. This might indicate that in Arabidopsis the proteolysis of Tim17-2 is developmentally regulated. It is also possible that during early stages of germination the seed-specific protein isoform Tim17-1 (Wang et al., 2014) is under proteolytic control of FTSH4. Furthermore, export of mitochondrially encoded components and assembly into the complexes is facilitated by the oxidase assembly translocase (OXA) located in the inner mitochondrial membrane (Kolli et al., 2018). Lately, Stiller et al. (2016) showed that in yeast OXA translocase is essential for importing not only mitochondrially, but nuclear-encoded proteins as well. Interestingly, OXA1-like protein has been identified as a potential interaction partner of FTSH4 (Opalinska et al., 2017b).

## Final conclusion

In this study we implemented for the first time the targeted proteomics approach, Multiple Reaction Monitoring, to quantify the protein-specific peptides of selected mitochondrial proteins of Arabidopsis seeds, from which the isolation of organelles is not possible. By applying MRM, we provided valuable information about the abundance dynamics of OXPHOS subunits and other mitochondrial membrane proteins in total protein samples from *A. thaliana* wild-type and *ftsh4* dry and germinating seeds. We showed that during germination the lack of the FTSH4 protease causes a diminished biogenesis of the oxidative phosphorylation system along with an increased abundance of AOX1A, which correlates with delay in germination of *ftsh4* seeds. The changes in OXPHOS and AOX1A protein levels were associated with a lower respiration rate, lower cytochrome pathway activity and higher activity of alternative oxidase. Furthermore, the observed changes in the abundance of the OXPHOS subunits occurred at the post-transcriptional level while the changes in the AOX1A abundance were caused at least partially by an increase in its transcript level. In summary, by the implementation of an integrative approach combining targeted proteomics, quantitative transcriptomics, and physiological studies we have shown an involvement of the mitochondrial FTSH4 protease in the biogenesis of the oxidative phosphorylation system during germination of Arabidopsis seeds.

## Author contributions

MH-C and HJ designed the study. MH-C, DD, and MK-O performed the experiments. All authors analyzed the data. MH-C wrote the manuscript with contribution from HJ.

### Conflict of interest statement

The authors declare that the research was conducted in the absence of any commercial or financial relationships that could be construed as a potential conflict of interest.
