# Supplementary material for: Targeted Proteomics Approach Toward Understanding the Role of the Mitochondrial Protease FTSH4 in the Biogenesis of OXPHOS During Arabidopsis Seed Germination
Source: Front Plant Sci. 2018 Jun 15;9:821. doi: 10.3389/fpls.2018.00821 (PMC6014109; doi:10.3389/fpls.2018.00821)
Supplement: Supplementary file 1 [file Table_1.PDF]

Supplemental Table S1. List of antibodies used in this study.

Antibody	Source	Antibody Type	Dilution
FTSH4	Agrisera (AS07 205)	polyclonal	1:1000
NAD9	kind gift from Jose M. Gualberto and Geraldine Bonnard, CNRS, Strasbourg, France	polyclonal	1:5000
RISP	kind gift from Monika Murcha, University of Western Australia, Australia	polyclonal	1:1000
AOX1/2	Agrisera (AS 04 054)	polyclonal	1:1000
Tim17-2	kind gift from Monika Murcha, University of Western Australia, Australia	polyclonal	1:1000
