# Supplementary material for: Targeted Proteomics Approach Toward Understanding the Role of the Mitochondrial Protease FTSH4 in the Biogenesis of OXPHOS During Arabidopsis Seed Germination
Source: Front Plant Sci. 2018 Jun 15;9:821. doi: 10.3389/fpls.2018.00821 (PMC6014109; doi:10.3389/fpls.2018.00821)
Supplement: Supplementary file 2 [file Table_2.PDF]

Supplementary Table S2. Primer pairs used for qRT-PCR.
AGI, Arabidopsis Genome Initiative Identifier (TAIR).

AGI	Gene	Forward primer	Reverse primer
**Reference gene**			
At2g20000	*HBT*	CGACTGGTTCTATGGCTTCC	CCTTCTTCCGAGTGTCCTAAG
**OXPHOS**			
**Complex I**			
AtMg00510	*nad7*	TGGCTCCAATGTCTTGGCTGTTG	CAATAGATCGCCCTTGTCGTTCAC
At5g37510	*NAD75*	TCGGTGTATCCAGTGTACAAGGTG	CCAGAGAGTTCACTGGTCATAAGC
At1g47260	*CA2*	CCGTCGAACGAATTAAGGAAGGC	ACCATACAAAGCAGGATTGGTCTC
**Complex II**			
At5g66760	*SDH1-1*	TCAAAGGAAGTGACTGGCTAGGTG	CGAGAGAAAGGCAGCCCATAATTC
At5g40650	*SDH2-2*	GATTGAATCTGGATCTAA	TTCATACATTCCATCAAG
At5g65165	*SDH2-3*	CATGGTTAAAGACGAGGAAGC	CCACCAATAAGAAGGACATG
**Complex III**			
AtMg00220	*cob*	TGGTATTTCCTACCGATCCATGCC	ACTGGTGCTATTGCGGCTACAC
At5g13440	*RIESKE*	ACCATAACCATGAGCGTTAC	CTTGCAGACATGCTCACAAT
**Complex IV**			
AtMg00160	*cox2*	CATCACATTTGACACCTG	CAGCCATTACTATCAAAGC
At2g47380	*COX5C-1*	GTCAAGGAGTTATTTATCG	AAGACAGAAATGGAAATC
**ATP synthase**			
AtMg01190	*atp1*	CTATTTACATTCCCGTCTC	GAATATAGGCCGATACGTC
At5g08670	*ATP2-1*	TAGTGTACGGACAAATGA	GTAGTAATTCGCTCTTGA
**Energy dissipating system**			
At3g22370	*AOX1A*	AGCATCATGTTCCAACGACGTTT	GCTCGACATCCATATCTCCTCTG
**Chaperone and membrane scaffold**			
At5g40770	*PHB3*	GTCACTAATCACCGTGCGAAG	GTTTCTGCTCAACAGCCCTTG
**Transport**			
At3g01280	*VDAC1*	TGGCTCCAATGTCTTGGCTGTTG	CAATAGATCGCCCTTGTCGTTCAC
At5g15090	*VDAC3*	CCCGATCACAAATCTGGAAAGGC	ACACCGGAGAAGTTGACAATCGG
