# Supplementary figures and images for: Targeted Proteomics Approach Toward Understanding the Role of the Mitochondrial Protease FTSH4 in the Biogenesis of OXPHOS During Arabidopsis Seed Germination
Source: Front Plant Sci. 2018 Jun 15;9:821. doi: 10.3389/fpls.2018.00821 (PMC6014109; doi:10.3389/fpls.2018.00821)

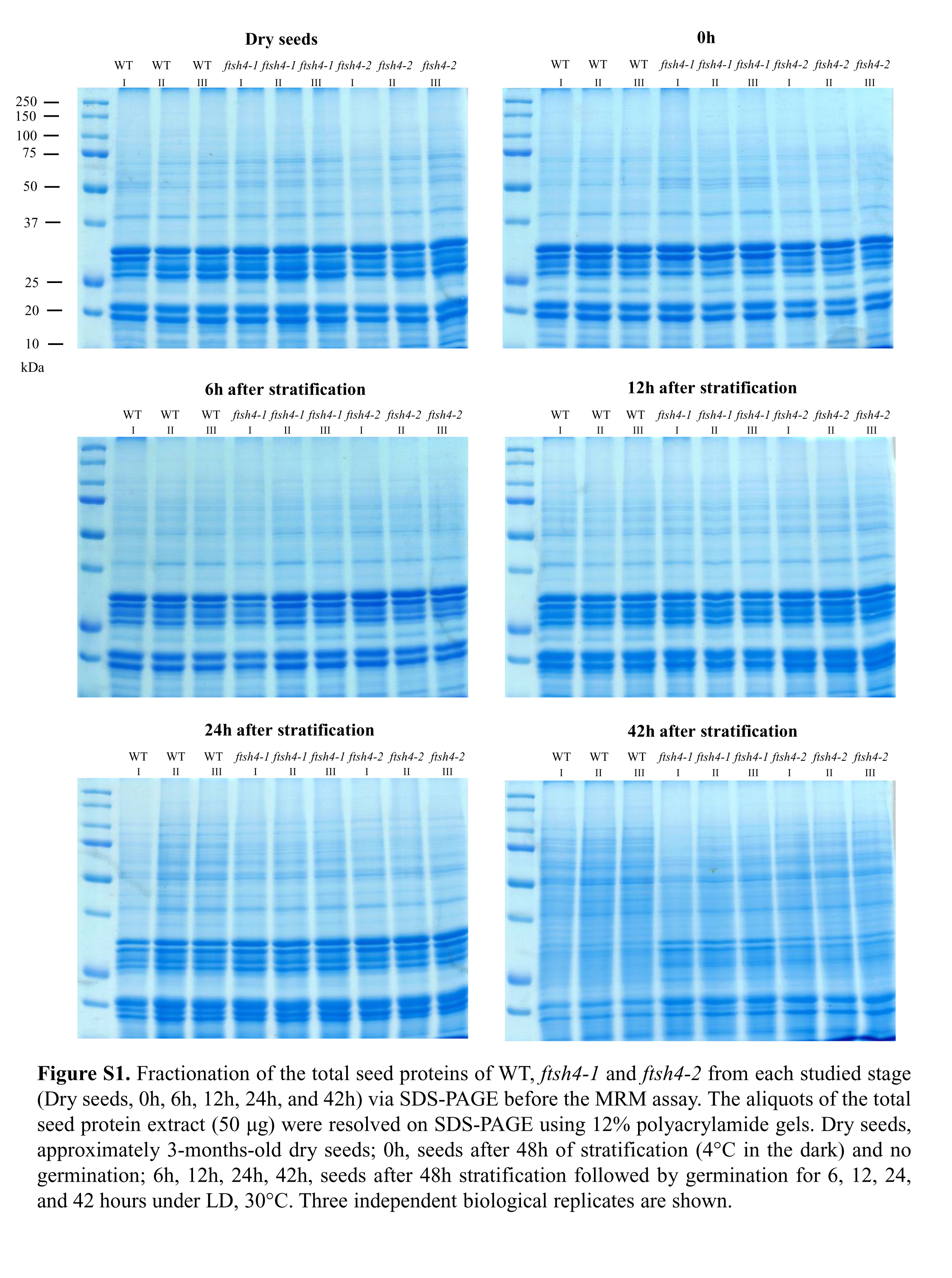

Supplement: Supplementary file 9 [file Image_1.TIF]

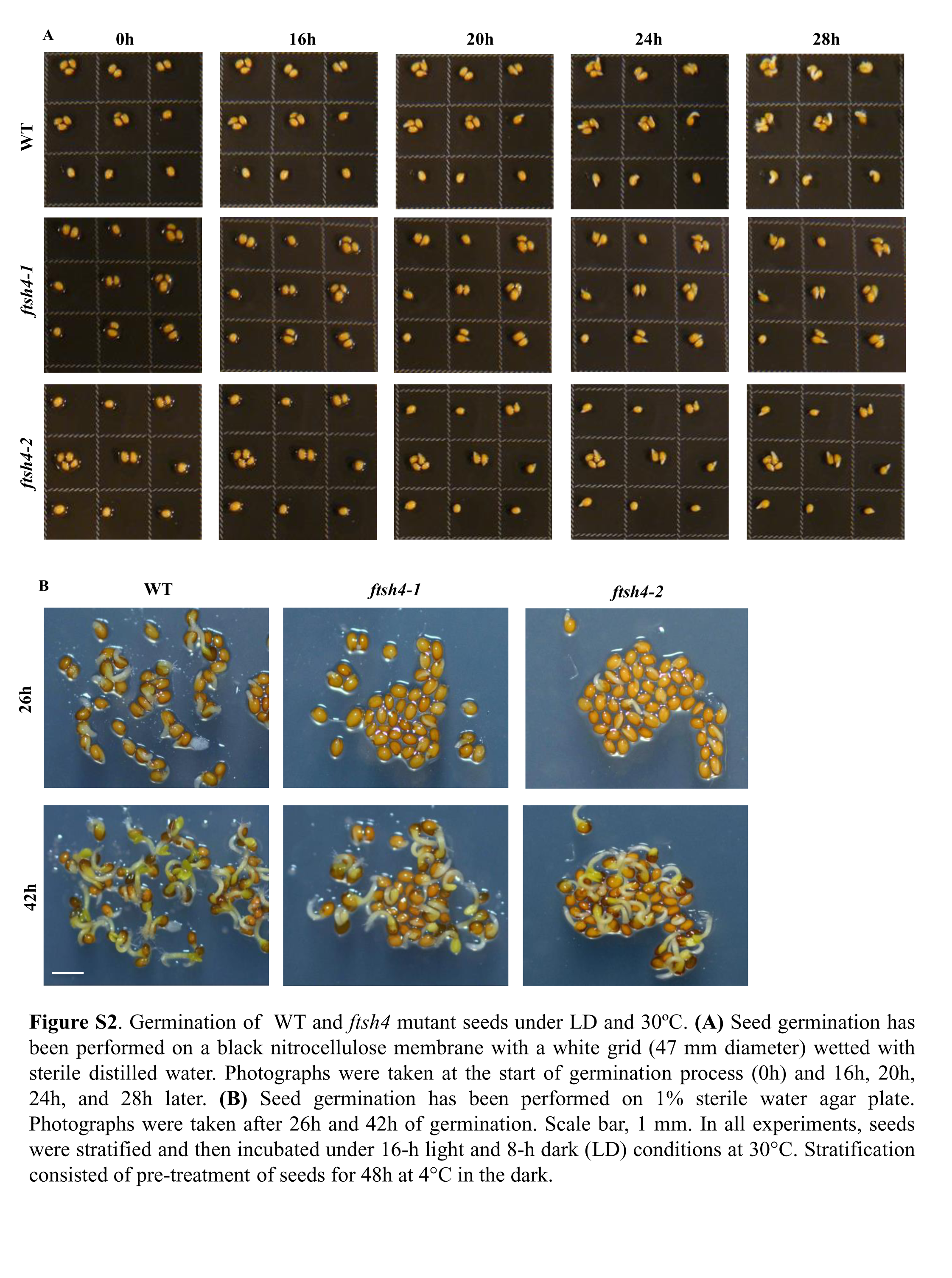

Supplement: Supplementary file 10 [file Image_2.TIF]

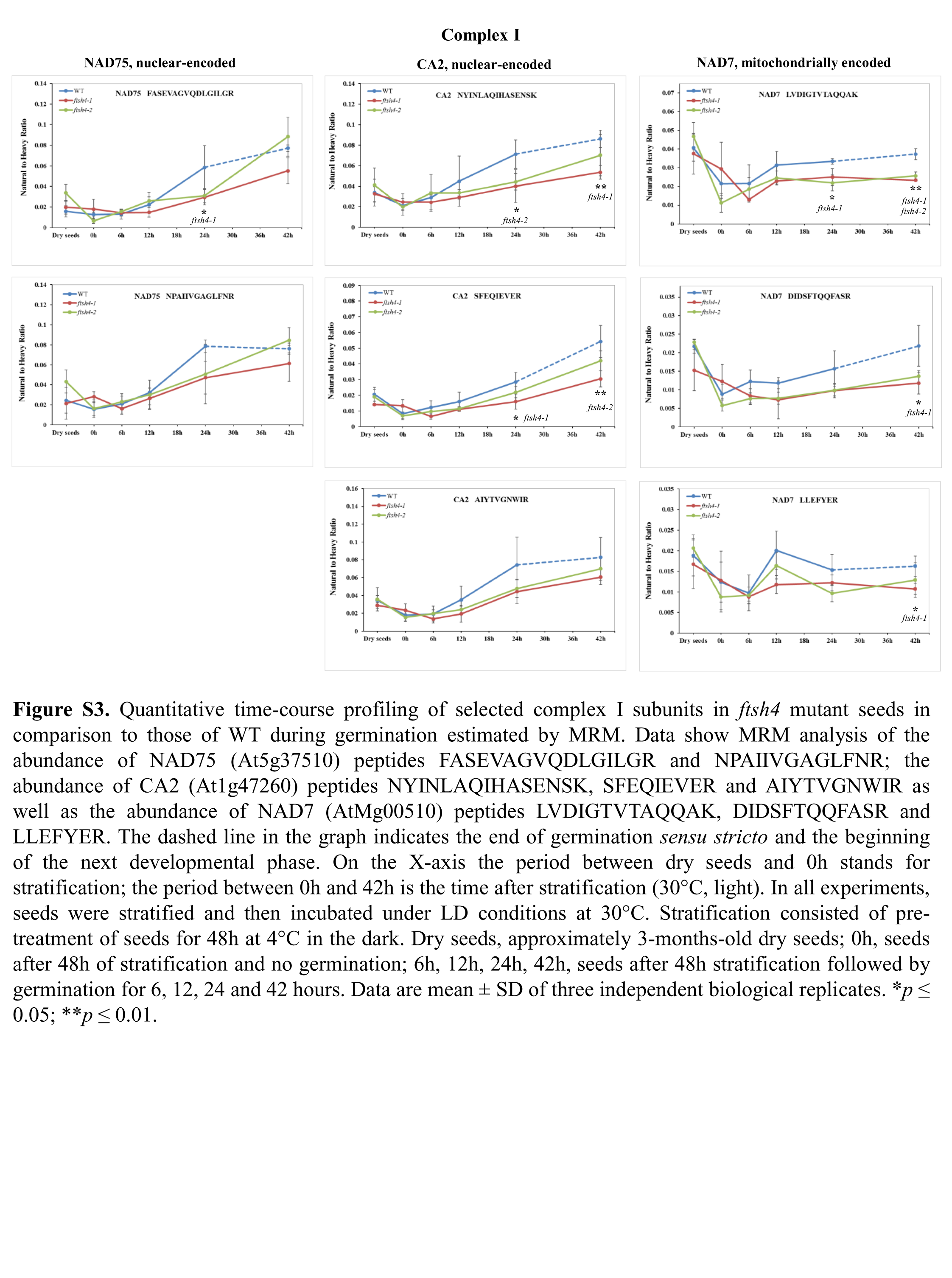

Supplement: Supplementary file 11 [file Image_3.TIF]

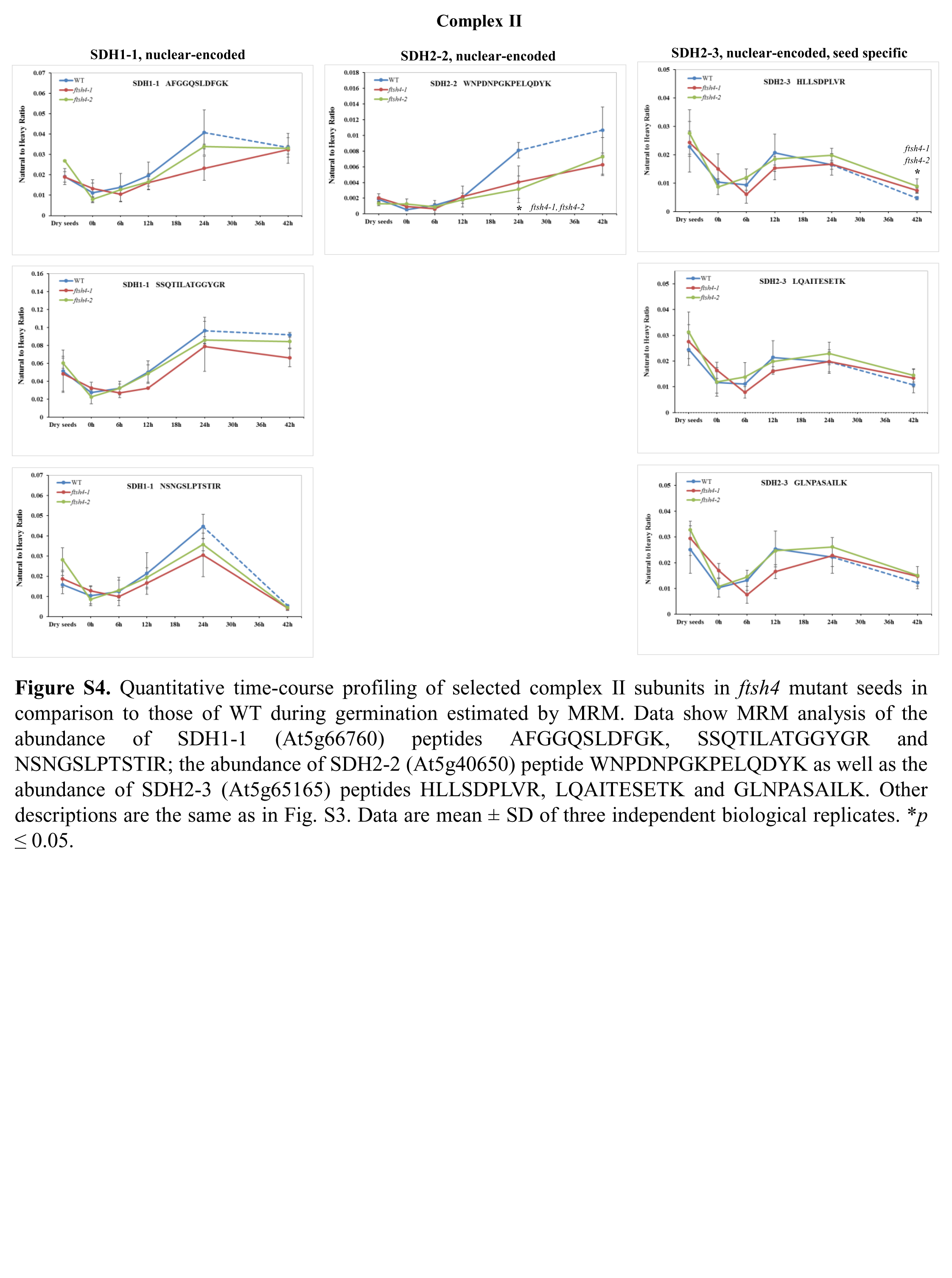

Supplement: Supplementary file 12 [file Image_4.TIF]

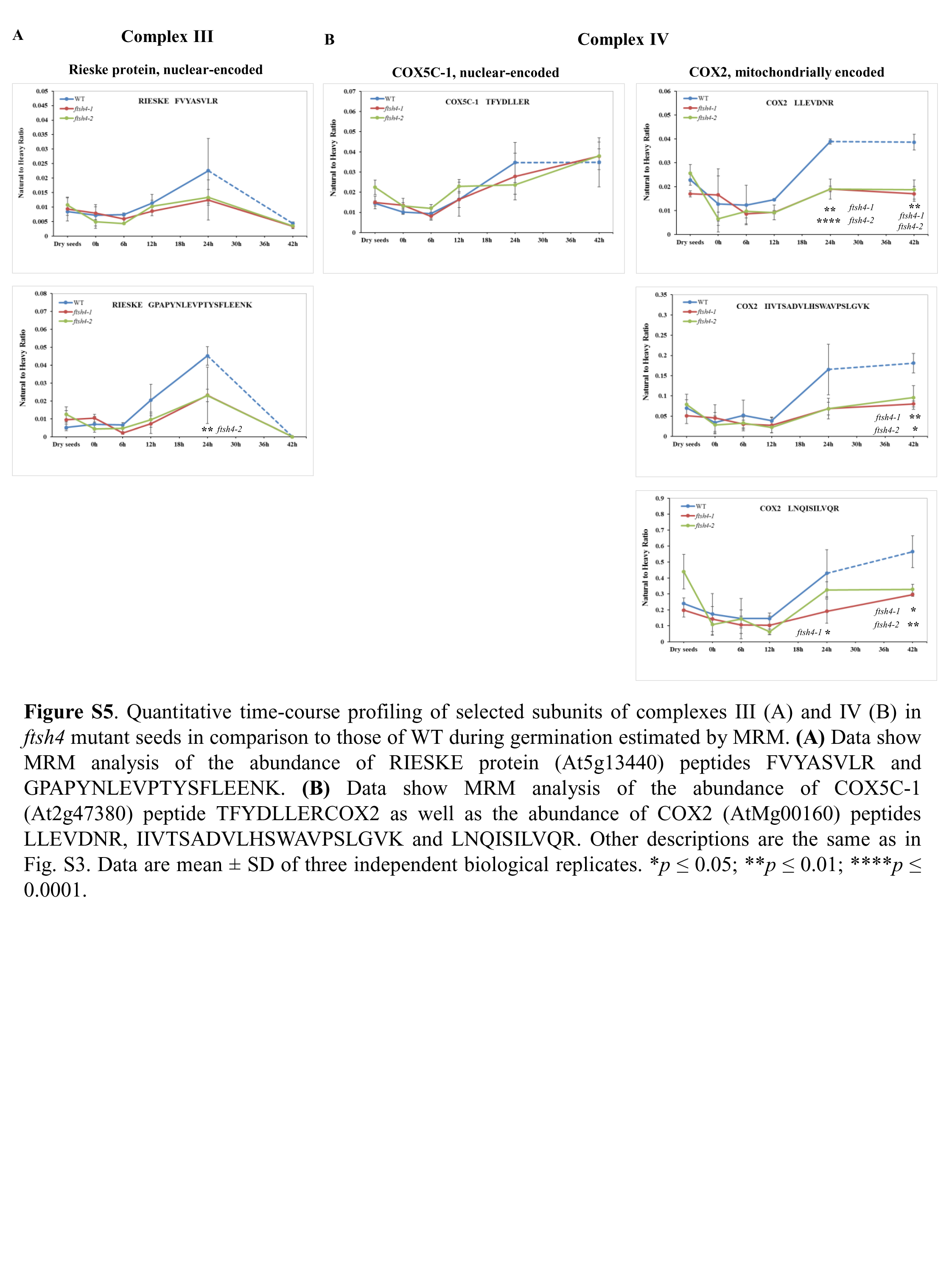

Supplement: Supplementary file 13 [file Image_5.TIF]

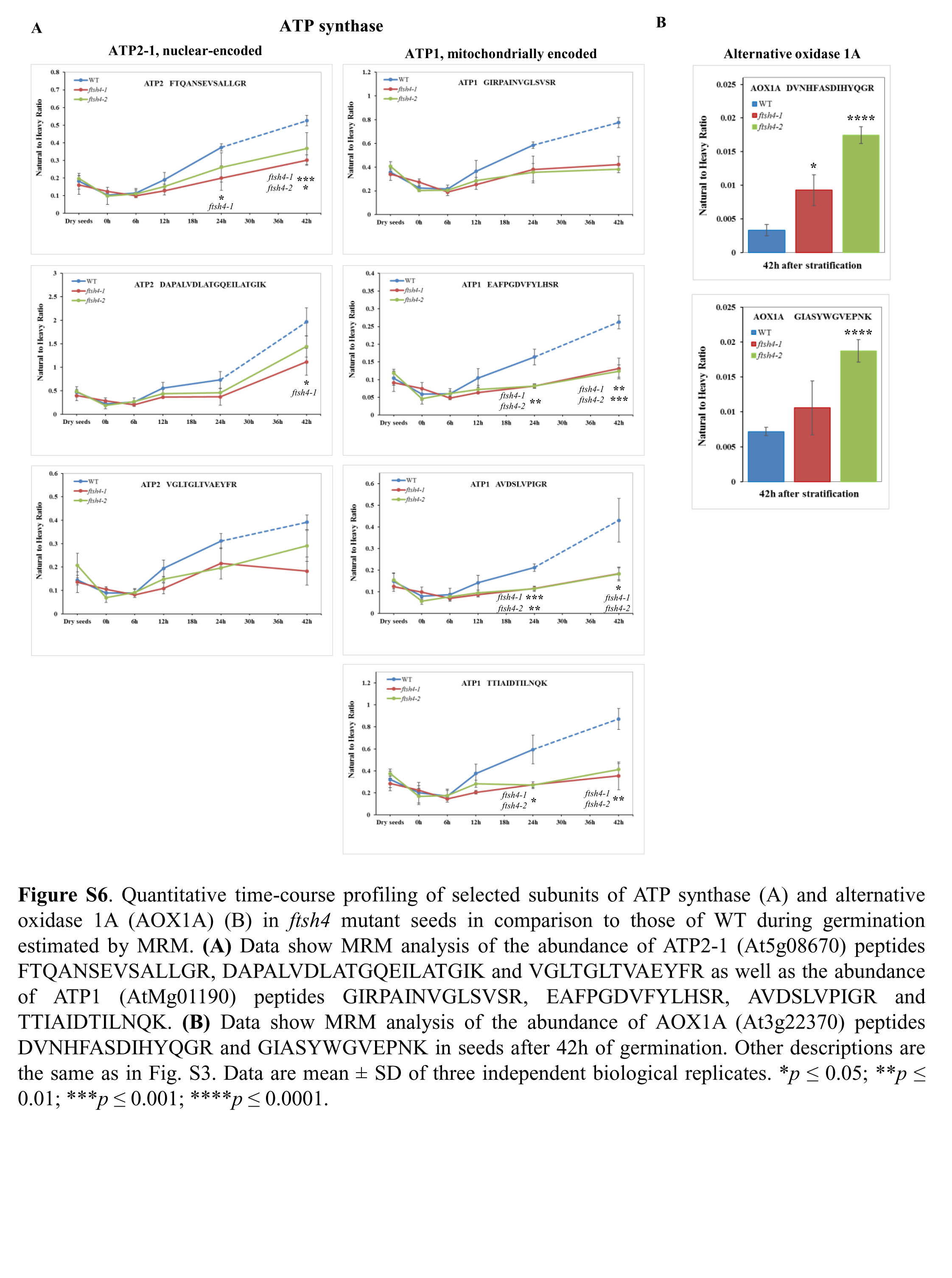

Supplement: Supplementary file 14 [file Image_6.TIF]

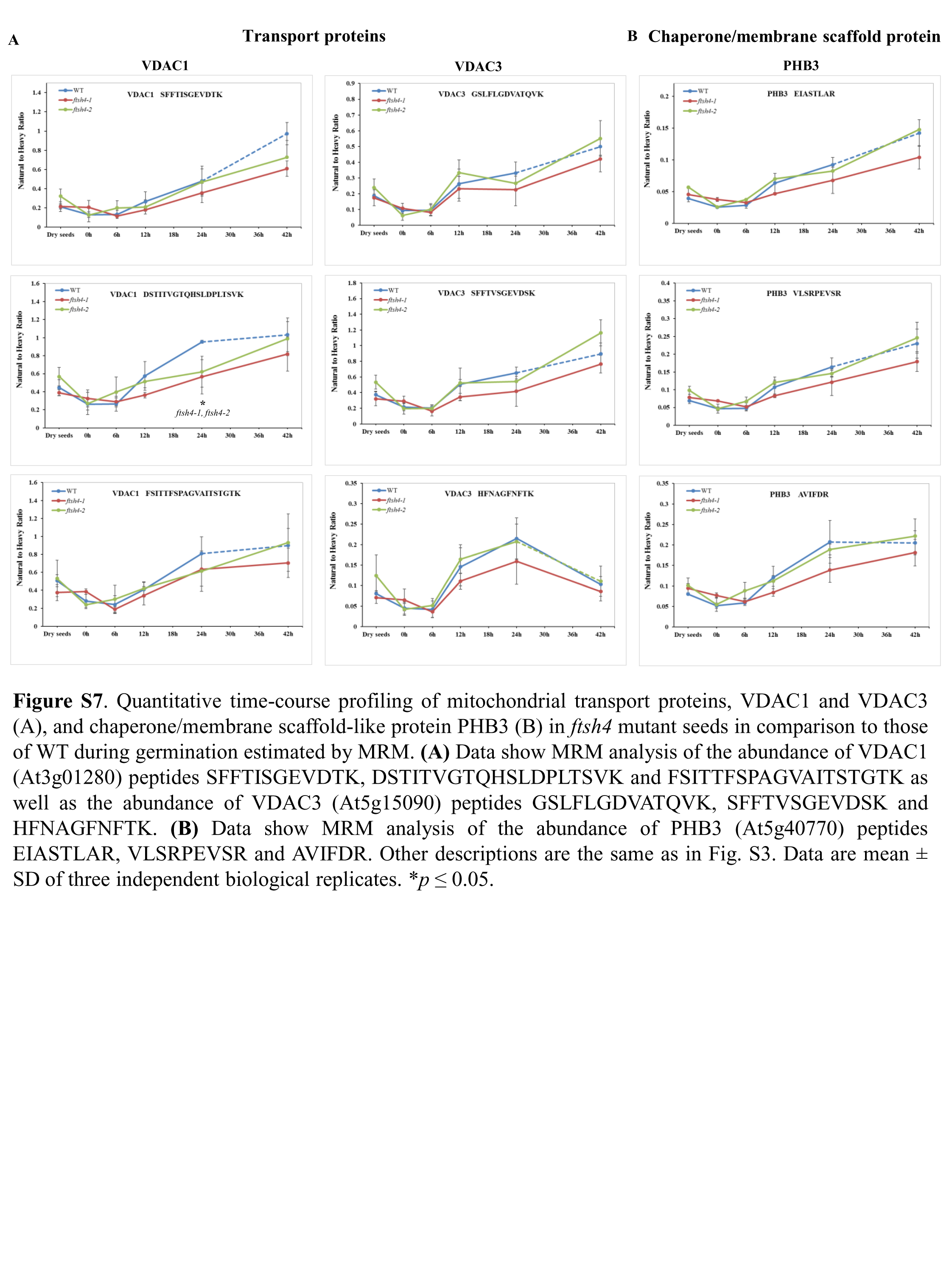

Supplement: Supplementary file 15 [file Image_7.TIF]

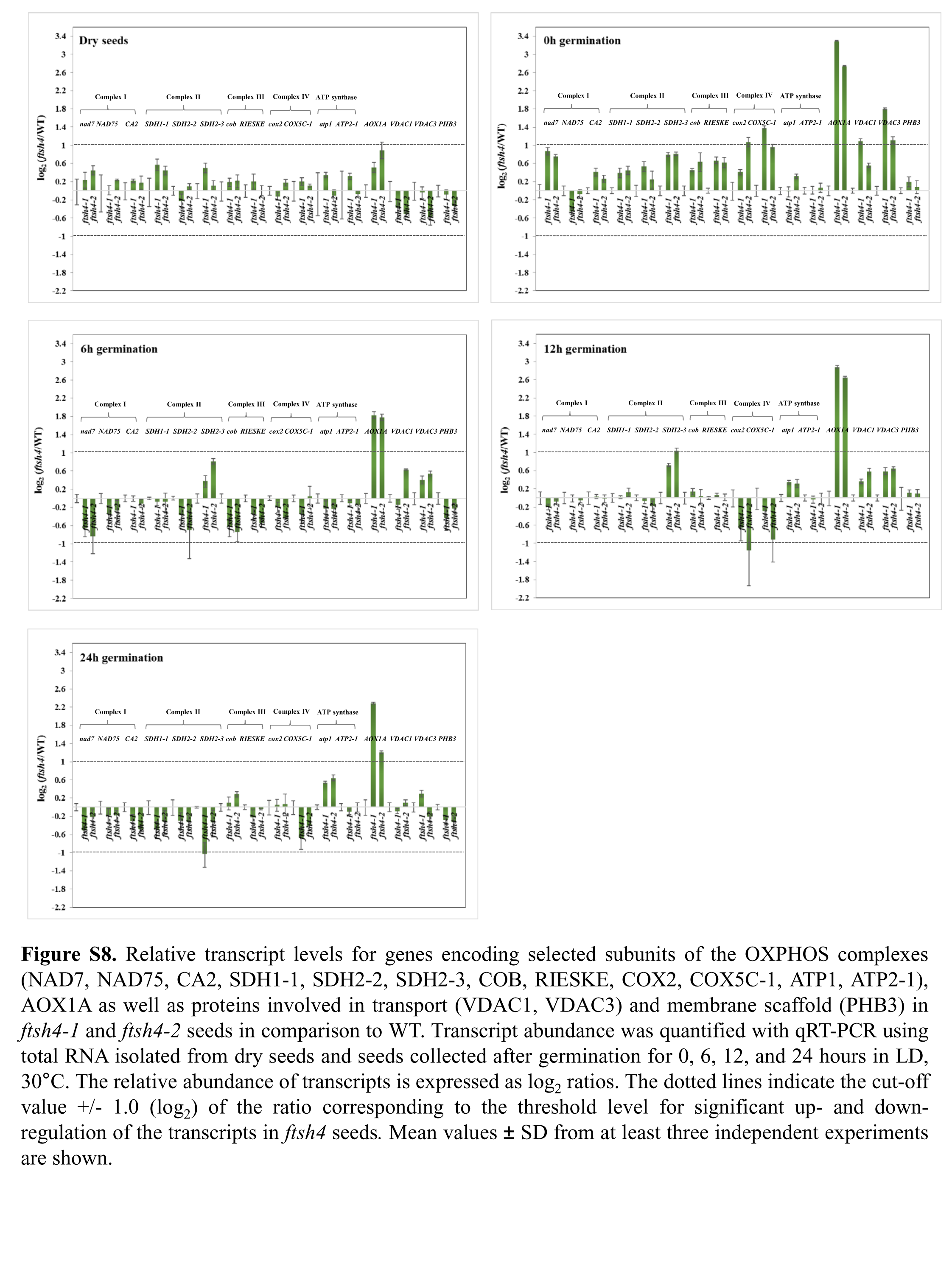

Supplement: Supplementary file 16 [file Image_8.TIF]
